# Exploiting a heterologous construction of the 3-hydroxypropionic acid carbon fixation pathway with mesaconate as an indicator in* Saccharomyces cerevisiae*

**DOI:** 10.1186/s40643-023-00652-5

**Published:** 2023-05-24

**Authors:** Shijie Xu, Weibo Qiao, Zuanwen Wang, Xiaoying Fu, Zihe Liu, Shuobo Shi

**Affiliations:** Beijing Advanced Innovation Center for Soft Matter Science and Engineering, College of Life Science and Technology, Beijing University of Chemical Technology, Beijing, 100029 China

## Abstract

The 3-Hydroxypropionic acid (3-HP) pathway is one of the six known natural carbon fixation pathways, in which the carbon species used is bicarbonate. It has been considered to be the most suitable pathway for aerobic CO_2_ fixation among the six natural carbon fixation pathways. Mesaconate is a high value-added derivative in the 3-HP pathway and can be used as a co-monomer to produce fire-retardant materials and hydrogels. In this study, we use mesaconate as a reporting compound to evaluate the construction and optimization of the sub-part of the 3-HP pathway in *Saccharomyces cerevisiae*. Combined with fine-tuning of the malonyl-CoA reductase (MCR-C and MCR-N) expression level and optimization of 3-Hydroxypropionyl-CoA synthase, the 3-HP sub-pathway was optimized using glucose or ethanol as the substrate, with the productions of mesaconate reaching 90.78 and 61.2 mg/L, respectively.

**Graphical Abstract:**

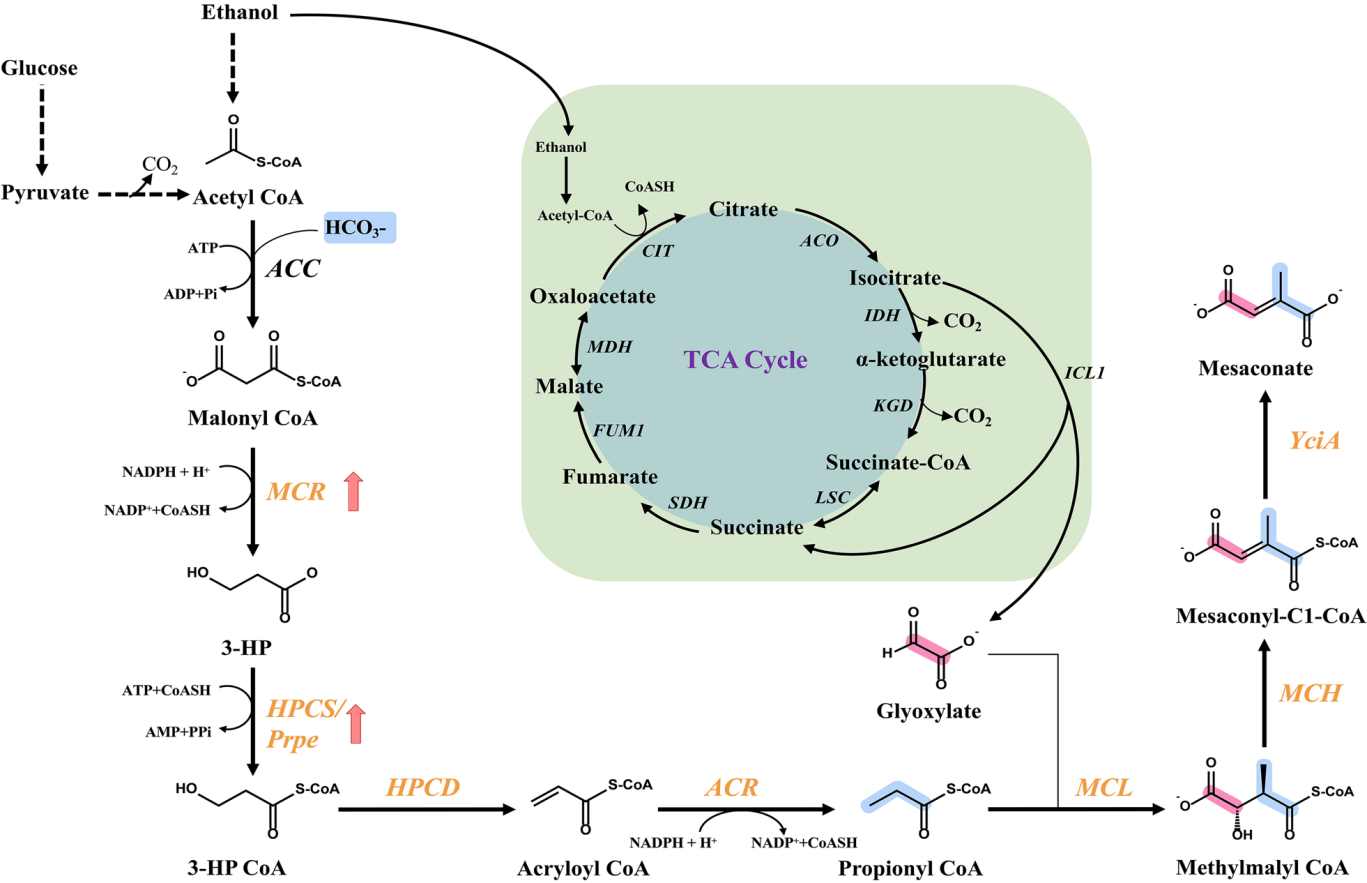

**Supplementary Information:**

The online version contains supplementary material available at 10.1186/s40643-023-00652-5.

## Introduction

Global warming caused by the greenhouse effect is one of the great challenges facing mankind, which will lead to melting of glaciers and rising sea levels (Glikson 2018). In 2020, the highest temperature in Antarctica reached 20.75 °C, a new record in history. The significant increase of the CO_2_ density in the atmosphere caused by fossil fuel combustion is the main reason for this phenomenon (Venkata Mohan et al. 2016). Therefore, the question on how to reduce the consumption of fossil fuels and develop alternative sustainable bioenergy has drawn great interest. Converting CO_2_ into biofuels or chemicals by engineered biological systems is one of the ways for CO_2_ resource utilization (Hu et al. 2019). The reconstruction of the CO_2_ fixation pathway within traditional microbial cell factories could recycle CO_2_ and reduce greenhouse gas emissions, which is of great significance for solving the shortage of fossil resources and alleviating the greenhouse effect.

There are six known natural carbon fixation pathways, including the Calvin (CBB) cycle, the 3-hydroxypropionic acid (3-HP) pathway, the Wood-Ljungdahl (WL) pathway, the reducing tricarboxylic acid cycle (rTCA) cycle, the dicarboxylic acid/4-hydroxybutyric acid (DC/HB) cycle and the 3-hydroxypropionic acid/4-hydroxybutyric acid (HP/HB) cycle. Among them, the 3-HP pathway has been considered to be the most suitable pathway for aerobic CO_2_ fixation (Liu et al. 2020). Unlike the CBB cycle, the WL pathway, the rTCA cycle and the DC/HB cycle, the carbon species used in this pathway is bicarbonate, whose concentration in equilibrium with air is much higher than CO_2_ in aqueous solution (Fuchs 2011). Therefore, the 3-HP pathway may be more efficient than those natural carbon fixation pathways using CO_2_ (Bar-Even et al. 2010). However, most of the research related to this pathway focused on the production of 3-HP (David et al. 2016; Liu et al. 2016).

Mesaconyl-C1-CoA is a key metabolic intermediate in the 3-HP pathway. The 3-HP pathway has been divided into four sub-pathways: (1) from acetyl-CoA to propionyl-CoA, (2) from propionyl-CoA to succinate, (3) from succinate to glyoxylate & acetyl-CoA, (4) from glyoxylate and propionyl-CoA to pyruvate and acetyl-CoA (Additional file 1: Fig. S1) (Mattozzi et al. 2013). In the sub-pathway (4), glyoxylate and propionyl-CoA are condensed into β-methylmalyl-CoA via MCL (malyl-CoA/beta-methylmalyl-CoA/citramalyl-CoA lyase), subsequently dehydrated to mesaconyl-C1-CoA by MCH (2-methylfumaryl-CoA hydratase), and then to pyruvate and acetyl-CoA catalyzed by MCT (2-methylfumaryl-CoA isomerase), MEH (3-methylfumaryl-CoA hydratase) and MCL. Almost all the intermediates are acyl-CoAs, which are difficult to quantify in cell extracts due to their easy hydrolysis and degradation properties. This makes it difficult to use these acyl-CoAs as an indicator compound to assess the efficiency of the assembled 3-HP pathway in the engineering host. However, by removal of the CoA moiety, mesaconyl-C1-CoA can generate its corresponding organic acid salt, mesaconate, under the reaction of the acyl-CoA thioester (Sonntag et al. 2014). When compared to 3-HP and propionate, mesoconate acts as the downstream product of 3-HP-CoA and propionyl-CoA. It serves as a crucial component when it comes to evaluating the construction and optimization of the 3-HP pathway in *S. cerevisia*e. As a result, mesaconate has been selected as the indicator compound utilized to evaluate the efficacy of the assembled 3-HP pathway in the engineering host.

*Saccharomyces cerevisiae* has long been the preferred metabolic engineering platform widely used in the refining of various biological products or chemicals, due to its numerous advantages (well characterized in genetics and physiology, available genome editing and gene expression tools) (Nielsen and Keasling 2016). At present, as a model organism for both basic and applied research, researchers have designed and expressed several carbon sequestration pathways in this heterotrophic industrial microorganism. For example, parts of the CBB cycle have also been heterogeneously reconstructed in *S. cerevisiae*, increasing the ethanol yields using CO_2_ as part of the carbon source (Guadalupe-Medina et al. 2013; Li et al. 2017). Gonzalez de la Cruz et al. (2019) enabled the reductive glycine (rGly) pathway in *S. cerevisiae* by overexpression of the endogenous enzymes. These works provide evidence for the potential modification of CO_2_ utilization in *S. cerevisiae*, which was also selected as the host in this work.

**Fig. 1:**
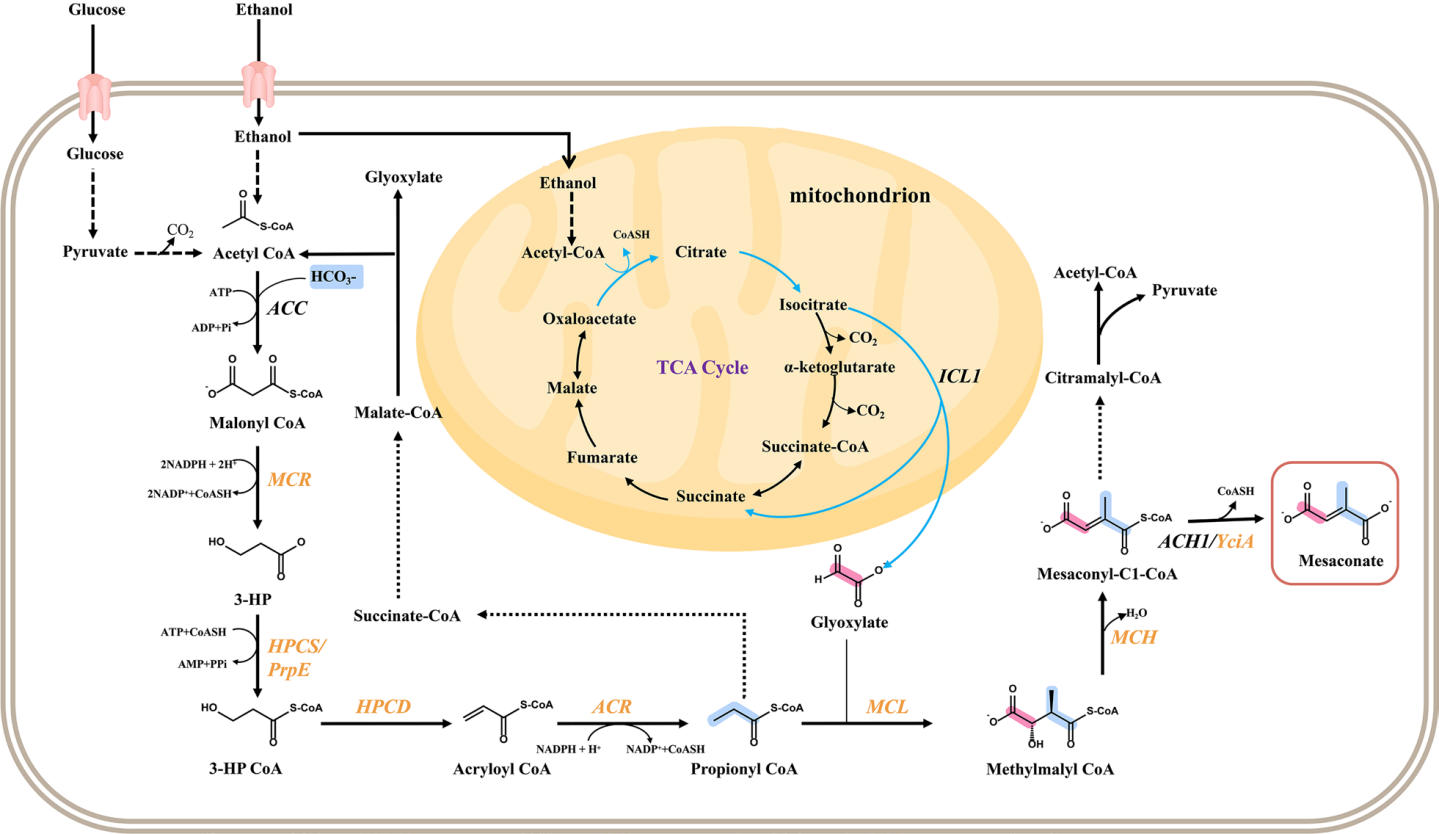
The designed sub-part of the 3-HP pathway in *S. cerevisiae.* The blue line indicates the glyoxylate cycle. Black font represents yeast endogenous enzymes, and yellow font represents exogenous enzymes. The dashed lines mean the conversion needs multi-step reactions. The blue color in chemical structures means the carbon atoms are from glyoxylate, and red color in chemical structures means the carbon atoms are from propionyl-CoA. *ACC* acetyl-CoA carboxylase, *MCR* malonyl-CoA reductase, *HPCS* 3-hydroxypropionyl-CoA synthase, *PrpE* propionyl-CoA synthetase, *HPCD* 3-hydroxypropionyl-CoA dehydratase, *ACR* acryloyl-CoA reductase, *MCL* malyl-CoA/beta-methylmalyl-CoA/citramalyl-CoA lyase, *MCH* 2-methylfumaryl-CoA hydratase, *ACH* acetyl-CoA hydrolase, *YciA* acyl-CoA thioester hydrolase

In this study, part of the 3-HP pathway was successfully constructed and optimized in *S. cerevisiae* using glucose or ethanol as the substrate (Fig. 1). Under both conditions, the production of mesaconate was used as an indicator compound for the optimization of the 3-HP sub-pathway and was adopted to evaluate the suitability of constructing the 3-HP pathway. After optimization of the 3-HP sub-pathway, the production of the mesaconate reporter reached 90.78 and 61.2 mg/L from glucose and ethanol, respectively. The detection of the produced mesaconate demonstrates the feasibility of constructing the 3-HP pathway in *S. cerevisiae*.

## Materials and methods

### Strains, plasmids and chemicals

*E. coli* DH5α was used for cloning and plasmid propagation. *S. cerevisiae* CEN.PK2-1D was used as the host for the construction of the 3-HP sub-pathway and mesaconate biosynthesis. The strains and plasmids used in this study are listed in Additional file 1: Tables S1 and S2. 3-HP was obtained from Sigma–Aldrich (St. Louis, MO, USA). Mesaconate and itaconate were purchased from Yuanye Biotechnology Co., Ltd. (Shanghai, China).

### Plasmids construction

The following genes were used to construct the 3-HP sub-pathway for the synthesis of mesaconyl-C1-CoA in CEN.PK2-1D, including genes encoding malonyl-CoA reductase (*Ca*MCR, uniport ID: Q6QQP7) from *Chloroflexus aurantacus*, 3-hydroxypropionyl-CoA synthase (*Ms*HPCS, uniport ID: A4YGR1) and acryloyl-CoA reductase (*Ms*ACR, uniport ID: A4YGN2) from *Metallosphaera sedula*, 3-hydroxypropionyl-CoA dehydratase (*St*HPCD, uniport ID: F9VNG3) from *Sulfolobus tokodaii*, propionyl-CoA synthetase (*SePrpE*, uniport ID: P55912) from *Salmonella enterica*, the malyl-CoA/beta-methylmalyl-CoA/citramalyl-CoA lyase (*Rs*MCL, uniport ID: A3PGR7) from *Rhodobacter sphaeroides*, and 2-methylfumaryl-CoA hydratase (*Ca*MCH, uniport ID: A9WC34; *Rs*MCH, uniport ID: Q3IZ78; *Hm*MCH, uniport ID: Q5V464) from *C. aurantacus*, *R. sphaeroides* and *Haloarcula marismortui.* The genes encoding acyl-CoA thioester hydrolase (*Ec*YciA, uniport ID: P0A8Z0) from *E. coli* was used to convert mesaconyl-C1-CoA to mesaconate. The genes encoding acetyl-CoA hydrolase (*ACH1*), 3-hydroxyisobutyryl-CoA hydrolase (*EHD3*) and peroxisomal acyl-CoA thioesterase (*TES1*) from *S. cerevisiae* were selected to verify mesaconyl-C1-CoA hydrolase function. Those genes were amplified or synthesized by TSINGKE (Beijing, China), and cloned into appropriate plasmids, respectively. For the combined expression of related genes, different gene expression cassettes were assembled into appropriate plasmids using ClonExpress® Ultra One Step Cloning Kit (Nanjing Vazyme Biotech Co., Ltd, China), and the primers used for fragments amplification are listed in Additional file 1: Table S3.

### Genomic manipulation by GTR-CRISPR

The GTR-CRISPR system (Zhang et al. 2019) was used for the genetic manipulation in CEN.PK2-1D. The sequences of gRNAs for the deletion of *ACH1*, *EHD3* and *TES1* were predicted at https://atum.bio/eCommerce/cas9/input. The expression cassettes were integrated at the XI-2, XI-3 and XII-1 sites (Jessop-Fabre et al. 2016). The primers used for integrations are listed in Additional file 1: Table S3.

### Fermentation and metabolite extraction

The transgenic CEN.PK2-1D cells were cultured with yeast extract peptone dextrose (YPD) medium or appropriate synthetic defined dropout liquid medium (SC) containing 2% glucose or 2% ethanol with 0.1% acetate, which could allow *Saccharomyces cerevisiae* to enter the logarithmic growth stage earlier (Additional file 1: Fig. S3). All yeast strains were cultured at 30 °C and 200 rpm in shake flasks.

For metabolite extraction, 200 mL of 0.5 mm glass beads, 30 μL of 12 M HCl and 10 μL of 0.2 g/L itaconate were added into 400 μL cell culture. After vortexing for 10 min, the mixture was incubated at 80 °C for 30 min and subsequently cooled to room temperature. Then 50 μL of NaCl saturated solution and 800 μL of ethyl acetate were added, and the samples were then vortexed for 10 min and centrifuged for 5 min at 5000 g. The organic phase was transferred into a new tube, and this extraction step was repeated once. The ethyl acetate extracts were vacuum-evaporated and derivatized with N-(tert-Butyldimethylsilyl)-N-methyl-trifluoroacetamide (MTBSTFA) at 80 °C for 30 min. 2 μL of the derivatized sample was subjected to GC–MS analysis.

### Promoter strength assay

The promoters of *TEF1*, *PGK1*, *TDH3*, *TPI1*, *QCR10*, *COX9*, *HXT1* and *NAT2* were amplified from the genomic DNA of *S. cerevisiae* CEN.PK2-1D. Primers used for promoter amplification are listed in Additional file 1: Table S3. The green fluorescent protein (GFP) was adopted for the determination of promoter strength. Plasmids containing *GFP* with different promoters mentioned above were transformed into *S. cerevisiae* CEN.PK2-1D. After culture for 72 h in SC-his medium that used 2% glucose or 2% ethanol (with 0.1% acetate) as the carbon source, the fluorescence intensity was measured by CytoFLEX (Backman Coulter, USA) for the quantification of GFP expression.

### HPLC and GC–MS analyses

3-HP was determined by HPLC with an LC-20AT instrument (Shimadzu, Kyoto, Japan) using an Aminex HPX-87H column (Bio-Rad, Hercules, USA). The column temperature was maintained at 60 °C using 5 mM H_2_SO_4_ as the mobile phase at a flow rate of 0.6 ml/min, and the signal was detected using a RI-101 refractive index detector.

Mesaconate was quantified using a GC/MS-QP2010 Ultra (Shimadzu, Kyoto, Japan) equipped with a HP-5 MS column (0.25 mm ID × 0.25 μm Film Thickness × 15 m Length). The column temperature was programmed at an initial temperature of 55 °C for 1 min, then increased to 115 °C at a rate of 30 °C/min and held for 15 min, then increased to 160 °C at a rate of 5 °C/min, then increased to 300 °C at a rate of 30 °C/min, and finally held at 300 °C for 15 min. The temperature of the injector was set at 270 °C. Helium was used as the carrier gas at a flow rate of 1 ml/min. The MS system was operated in full scan mode within the *m/z* range of 50–600.

## Results and discussions

### Construction of a 3-hp sub-pathway in *S. cerevisiae*

As shown in Fig. 1, a 3-HP sub-pathway was designed and constructed in *S. cerevisiae*, with the formation of mesaconate as the reporter metabolite. This sub-pathway covers reactions from acetyl-CoA to mesaconyl-C1-CoA in the 3-HP pathway (Additional file 1: Fig. S1).

**Fig. 2:**
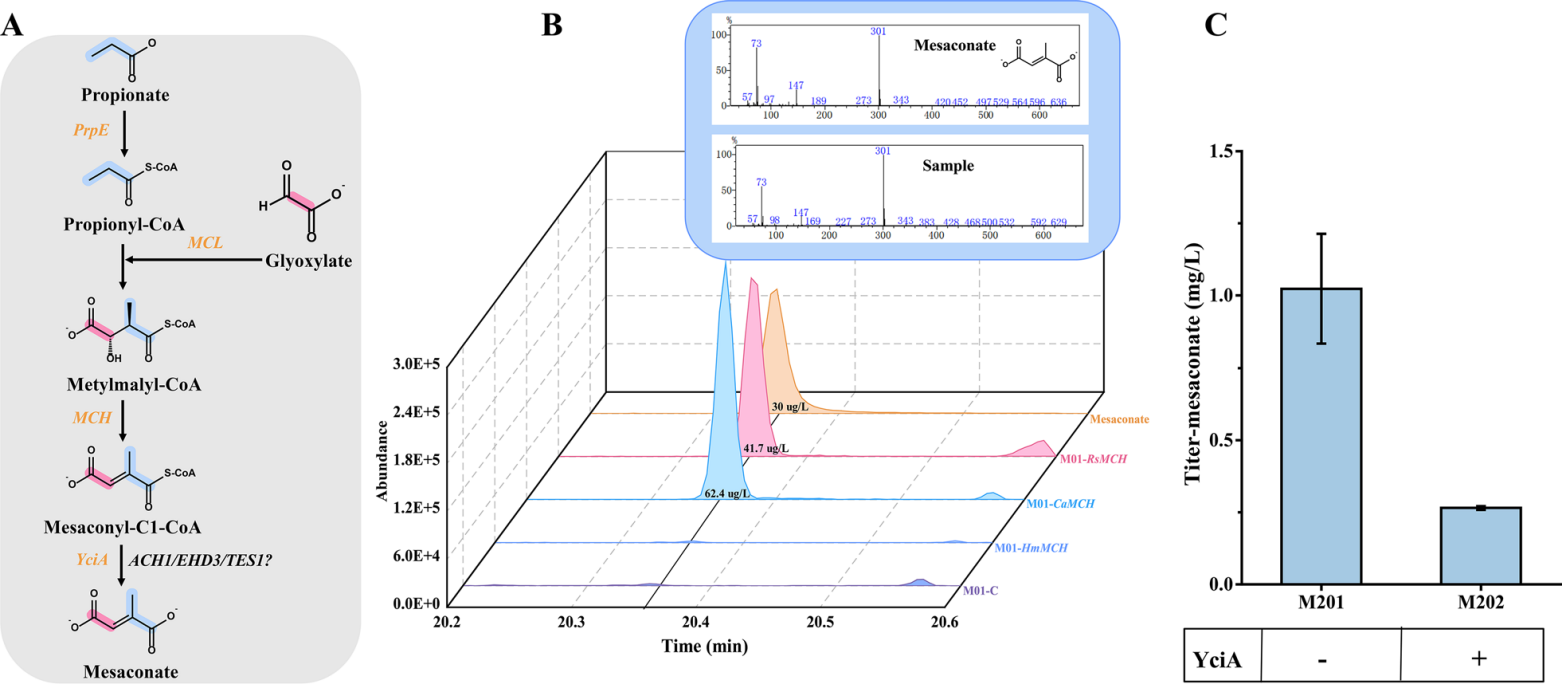
Construction of a 3-HP sub-pathway in *S. cerevisiae.* **A** Part of the 3-HP sub-pathway from propionate and glyoxylate. **B **GC–MS analysis of mesaconate produced from propionate and glyoxylate in engineered yeasts with various MCHs from different species. Strains were cultured with SC-dropout medium with 0.1% propionate and 0.1% glyoxylate. M01: CEN.PK 2-1D with the expression of *SePrpE*, *RsMCL* and *EcYciA*; M01-C: M01 with empty plasmid; M01-*CaMCH*: M01 with the expression of *CaMCH*; M01-*RsMCH*: M01 with the expression of *RsMCH*; M01-*HmMCH*: M01 with the expression of *HmMCH*. Mesaconate, the standard of mesaconate at 30 μg/L. **C** The yield of the reporter mesaconate from glucose. M201, strain M2 harboring the plasmid pSC-HIS-*MCR* for overexpressing *MCR*; M202, strain M2 harboring the plasmid pSC-HIS-*MCR*-*YciA* for overexpressing *MCR* and *YciA*. Strains were cultured with SC-dropout medium with 0.1% glyoxylate

In this 3-HP sub-pathway, we first overexpressed the MCL, MCH and an acyl-CoA thioester hydrolase (YciA) in CEN.PK 2-1D that composed the downstream reactions from propionyl-CoA to mesaconate (Fig. 1). To facilitate the confirmation of the function of MCL, MCH and YciA, propionate was added to the medium as well as additional expression of a propionyl-CoA synthetase (PrpE) to enable the supply of propionyl-CoA (Fig. 2A). When designing the pathway, the *Ec*YciA from *E.coli* and *Se*PrpE from *S. enterica* were chosen, since *Ec*YciA has been reported to be a non-specific thioesterase that is active on mesaconyl-CoA (Sonntag et al. 2014) and *Se*PrpE can convert propionate to propionyl-CoA (Ding et al. 2022). MCL is a multifunctional enzyme with both cleavage and condensation activities. We summarized the activities of MCLs from BRENDA (https://www.brenda-enzymes.info/index.php) (Table S4). Among them, *Rs*MCL has the highest ratio of condensation activity to cleavage activity and was adopted in our design. We chose and tested three MCHs, which are from *C. aurantacus*, *R. sphaeroides* and *H. marismortui*, respectively. As the result shown in Fig. 2B, mesaconate can be detected in strains that overexpressed *Ca*MCH from *C. aurantacus* or *Rs*MCH from *R. sphaeroides*, combined with the overexpression of *Se*PrpE, *Rs*MCL and *EcYciA*. The strain holding *Ca*MCH can produce more mesaconate, indicating higher efficiency of the constructed 3-HP sub-pathway. We integrated the preferred *RsMCL* and *CaMCH* into the genome of CEN.PK 2-1D, obtaining strain M1 for subsequent pathway construction.

Then, *MsHPCS* from *M. sedula*, *MsACR* from *M. sedula*, and *StHPCD* from *S. tokodaii* were integrated into M1 (obtaining strain M2). To constructed the whole 3-HP sub-pathway (Fig. 1), the plasmid pSC-HIS-*MCR* harboring *CaMCR* from *C. aurantacus* was transformed into M2 (obtaining strain M201), and the plasmid pSC-HIS-*MCR*-*YciA* harboring *CaMCR* and *Ec*YciA was transformed into M2 (obtaining strain M202). As shown in Fig. 2C, 1.02 mg/L of mesaconate was synthesized in M201 from glucose, suggesting that this whole 3-HP sub-pathway was successfully constructed in *S. cerevisiae*. Interestingly, the yield of mesaconate decreased to 0.26 mg/L after overexpressing an additional *EcYciA* in the engineered strain M202. We speculate that the acyl-CoA thioester hydrolase also can hydrolyze 3-hydroxypropionyl-CoA, thereby inhibiting the metabolic flux of 3-HP to mesaconate. In the subsequent optimization work, we did not overexpress the additional *Ec*YciA in the engineering strains.

### Evaluation and characterization of acyl-CoA hydrolases in the 3-HP sub-pathway

**Fig. 3:**
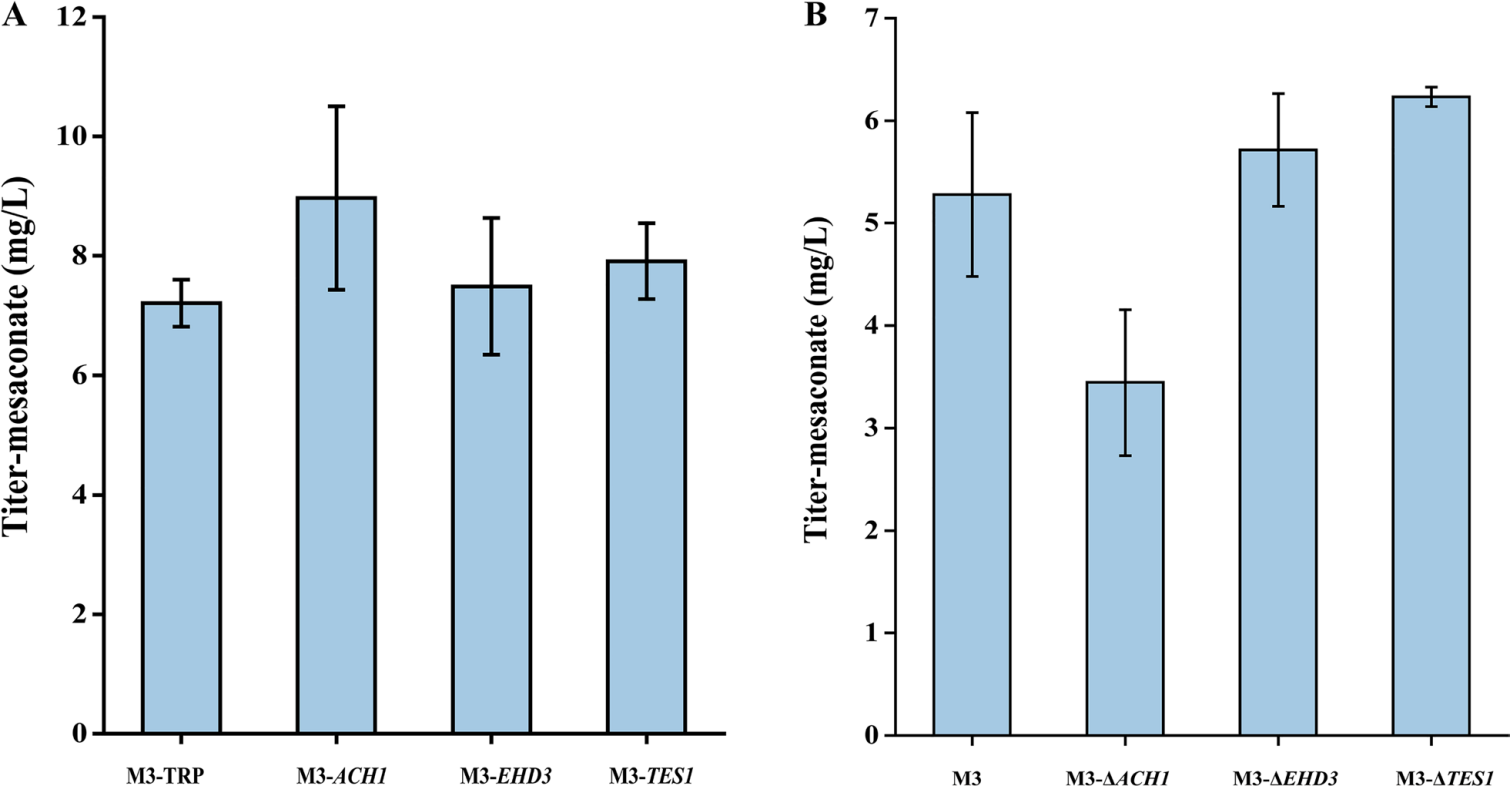
Functional validation of endogenous acyl-CoA hydrolases/transferases in yeast. **A** Production of mesaconate in yeast M3 with the overexpression of *ACH1*, *EHD3* and *TES1*. M3-TRP: M3 harboring the empty plasmid pSC-TRP; M3-*ACH1*: M3 harboring the plasmid pSC-TRP-*ACH1* for overexpressing *ACH1*; M3*-EHD3*: M3 harboring the plasmid pSC-TRP-*EHD3* for overexpressing *EHD3*; M3*-TES1*: M3 harboring the plasmid pSC-TRP-*TES1* for overexpressing *TES1*. **B** Production of mesaconate in yeast M3 with the knockout of *ACH1*, *EHD3* and *TES1*; M3-*ΔACH1*: deletion of *ACH1* in M3; M3*-ΔEHD3*: deletion of *EHD3* in M3; M3*-ΔTES1*: deletion of *TES1* in M3. Strains were cultured with SC-dropout medium with 0.1% propionate and 0.1% glyoxylate

It should be mentioned that mesaconate can also be detected in the absence of *Ec*YciA (Fig. 2C). We speculate that the endogenous acyl-CoA hydrolases in yeast may play a role in converting mesaconyl-C1-CoA to mesaconate (Fig. 2A). Here, genes encoding acetyl-CoA hydrolase (*ACH1*), 3-hydroxyisobutyryl-CoA hydrolase (*EHD3*) and peroxisomal acyl-CoA thioesterase (*TES1*) from *S. cerevisiae* were selected to verify the function of mesaconyl-C1-CoA hydrolase, since acyl-CoA hydrolase or thioesterase may have substrate promiscuity and showed hydrolase activity to broad substrates (Sonntag et al. 2014). We overexpressed and knocked out these three genes in M3 (*SePrpE* was integrated into M1), respectively. As shown in Fig. 3A, the accumulation of mesaconate increased by 24.4% after the plasmid-based overexpression of *ACH1* in M3. In contrast, mesaconate was significantly reduced when knocking out *ACH1* in M3 (Fig. 3B). The results showed the thioesterase encoded by *ACH1* has mesaconyl-CoA hydrolase activity.

### Optimization of the MCR expression in the 3-HP sub-pathway

MCR has been reported as the key enzyme in the 3-HP pathway, catalyzing the reduction of malonyl-CoA to 3-HP (Son et al. 2020). In this section, we tested whether the efficiency of 3-HP pathway can be improved by increasing the accumulation of 3-HP via optimizing of MCR. There are two functional domains in MCR, which catalyzes malonyl-CoA to 3-HP through a two-step NADPH-dependent reduction (Hügler et al. 2002; Liu et al. 2013). Liu et al. (2013) made an intriguing discovery which showed that MCR can be split into two segments, namely MCR-C (amino acids 550–1219) and MCR-N (amino acids 1–549). MCR-C is involved in catalyzing malonyl-CoA to malonate semialdehyde, while MCR-N transforms malonate semialdehyde to 3-HP. The expression of these two fragments had an important impact on 3-HP production. It also reported that even when both genes were codon-optimized and controlled by the same promoter, MCR-N had a higher expression level than MCR-C (Liu et al. 2013). This enzyme activity discrepancy has the potential to affect the overall production of 3-HP.

**Fig. 4:**
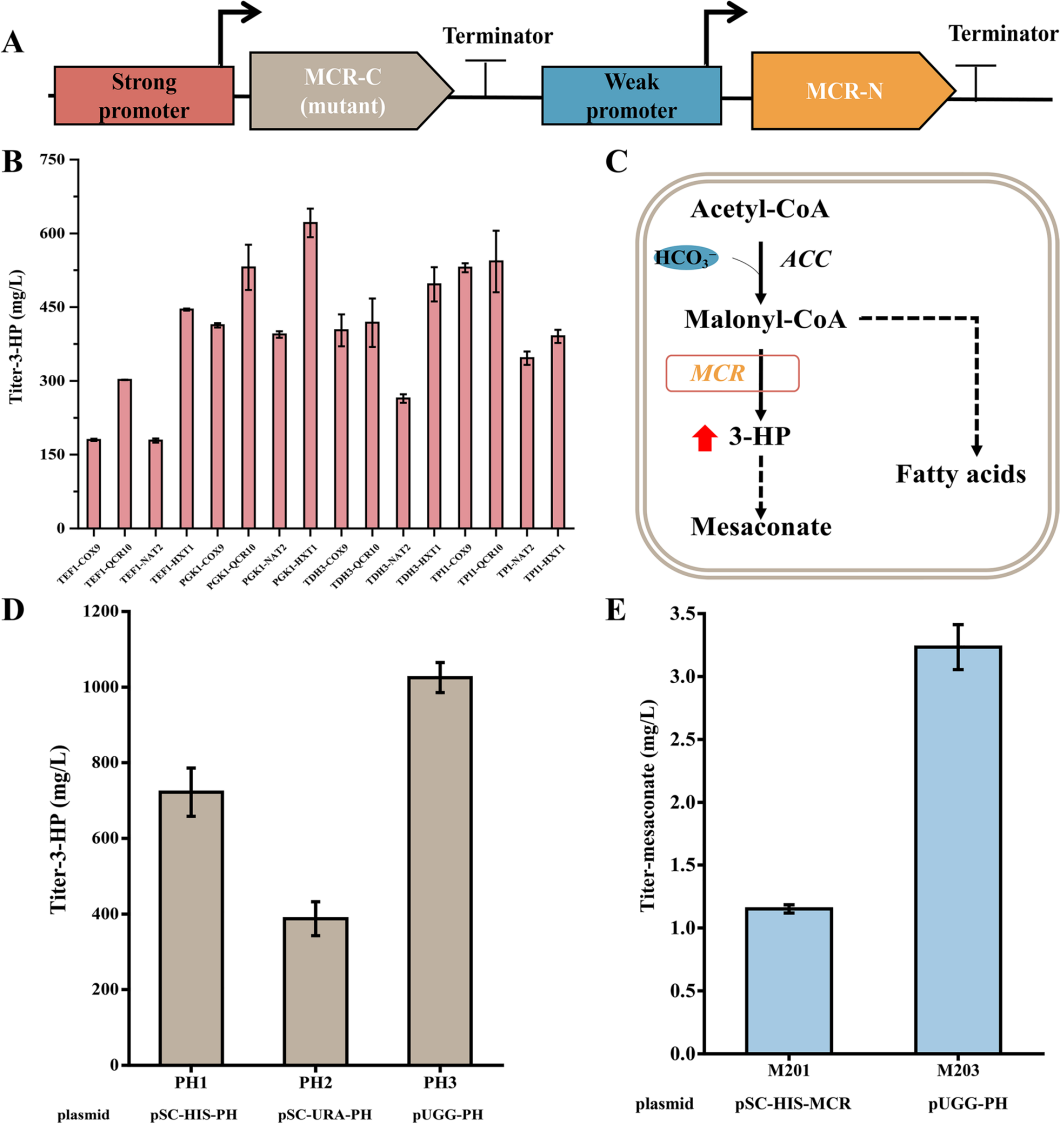
Optimization of MCR for the increased 3-HP accumulation. **A** Promoter optimization strategy for the expression of MCR. **B** 3-HP production in engineered yeasts from glucose by expression of MCR-C and MCR-N under control by different promoter combinations. **C** The competing synthetic pathway of 3-HP and fatty acid. Intracellular fatty acid synthesis competes with the 3-HP sub-pathway for catalyzing malonyl-CoA. **D** 3-HP production in engineered yeasts from glucose by expression of MCR using different plasmids, including 2 μ-based plasmid pSC-HIS-PH, 2 μ-based plasmid pSC-URA-PH, and ultrahigh copy number plasmid pUGG-PH. **E** The yield of the reporter mesaconate from glucose. M201, strain M2 harboring the plasmid pSC-HIS-*MCR* for overexpressing *MCR*; M203, strain M2 harboring the plasmid pUGG-PH for overexpressing *MCR*. Strains were cultured with SC-dropout medium with 0.1% glyoxylate

To solve this problem, Liu et al. (2016) significantly increased the 3-HP production by fine tuning of the expression level of MCR-N combined with directed evolution of MCR-C (mutation in N940V, K1106W and S1114R). In this study, the two functional domains were expressed under promoters of different strengths, respectively. Promoters of *PGK1*, *TEF1*, *TDH3*, *TPI1, HXT1*, *QCR10*, *COX9* and *NAT1* are selected to fine tune the expression level. We determined the ability of these promoters under glucose by measuring the expression level of GFP (Additional file 1: Fig. S2). Stronger promoters, such as PGK1p, TEF1p, TDH3p and TPI1p, were used for expression of MCR-C_*mut*_ (N940V, K1106W and S1114R) and weaker promoters, such as HXT1p, QCR10p, COX9p and NAT2p, were adopted for expression of MCR-N (Fig. 4A). As shown in Fig. 4B, the combination of *PGK1p-MCR-C*_*mut*_ and *HXT1p-MCR-N* produced the highest yield of 3-HP in glucose. When the expression level of MCR-N is lower than that of MCR-C_*mut*_, it helps to improve 3-HP production. However, insufficient expression of the MCR-N protein may not effectively reduce the MSA produced by MCR-C_*mut*_, thus limiting 3-HP biosynthesis. The results show that a balanced expression level between MCR-C_*mut*_ and MCR-N is an essential factor for 3-HP production.

Since cellular intracellular fatty acid synthesis usually consumes malonyl-CoA, competing with the 3-HP pathway (Fig. 4C). To further drive the malonyl-CoA flux into the 3-HP pathway instead, we used an ultrahigh copy number plasmid pUGG to replace the typical 2μ-based plasmid for the expression of 3-HP formation genes. The ultrahigh copy number plasmid with a truncated promoter for the auxotrophic maker gene could reach ∼200 copies per cell, which is much higher than 10–40 copies of 2μ-based plasmid (Qin et al. 2020). After changing the copy number, the 3-HP accumulation increased to 1025 mg/L at shaker flask (Fig. 4D). Currently, considerable progress has been achieved in yeast-based 3-HP production via the MCR pathway (Chen et al. 2014, 2017; Kildegaard et al. 2016; Li et al. 2015; Maury et al. 2018; Yu et al. 2022). Many of the strategies implemented utilized incremental precursor and co-factor supply to enhance the production of 3-HP. In particular, Yu et al*.* optimized the expression of MCR in a super yeast chassis with sufficient supply of precursor malonyl-CoA and cofactor NADPH, greatly improving 3-HP yield (Yu et al. 2022). The result demonstrated that optimizing MCR expression is an effective strategy to increase the efficiency of the 3-HP pathway in yeast.

To test the effect of optimization strategy of MCR on the 3-HP sub-pathway, we transformed the ultrahigh copy number plasmid pUGG-PH into M2, obtaining strain M203. Indeed, the yield of the reporter mesaconate was increased to 3.23 mg/L, a 1.8 folds increase compared to that of the initial strain M201 (Fig. 4E). However, in contrast to the strong concentration of 3-HP, the yield of mesaconate remains notably low, leading us to presume that the hindrance lies in the reactions from 3-HP to mesaconate. The process of further reductive conversion of 3-HP to mesaconate demands six enzymatic steps, and the initial step is the activation of 3-HP to its CoA ester (3-HP-CoA). Therefore, our next objective is to evaluate whether the efficiency of the 3-HP sub-pathway can benefit from the selection of 3-hydroxypropionyl-CoA synthase.

### Overexpression of PrpE to increase the efficiency of the 3-HP sub-pathway

The 3-hydroxypropionyl-CoA synthase (HPCS) from *M. sedula* and propionyl-CoA synthetase (PrpE) from *S. enterica* were used in this project. Both HPCS and PrpE are multifunctional acyl-CoA synthases, belonging to propionate-CoA ligase (EC 6.2.1.17), have a rather broad substrate spectrum. It was reported that HPCS can use 3-HP, propionate, acrylate, acetate, and butyrate as substrates (Alber et al. 2008) and PrpE can catalyze acyl substrates, such as propionate, acrylate, acetate and butyrate, into corresponding acyl-CoA (Horswill and Escalante-Semerena 2002). Hence, we assume that PrpE can also take 3-HP as the substrate, similar to HPCS.

**Fig. 5:**
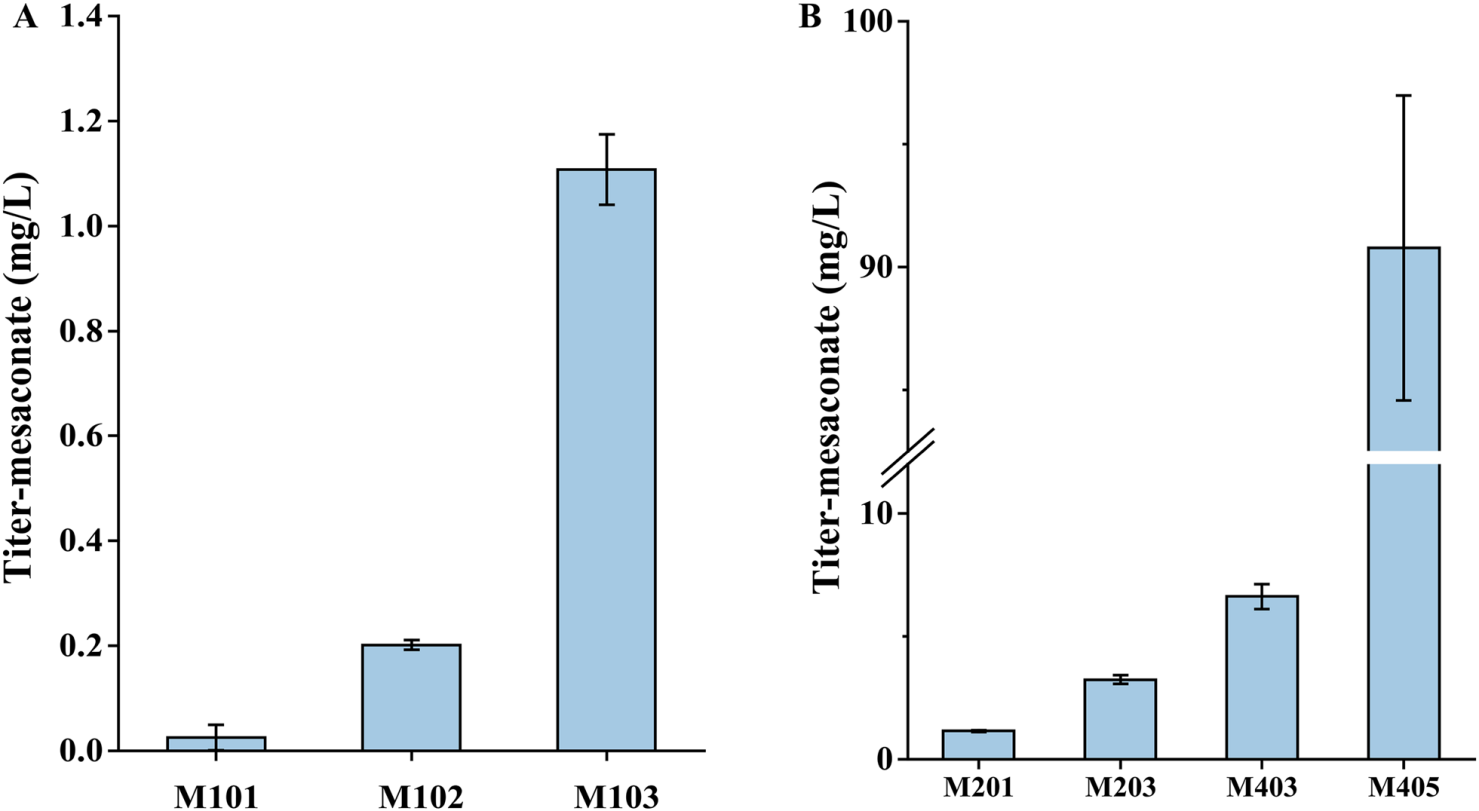
Optimization of the 3-HP sub-pathway in *S. cerevisiae*. **A** Evaluation of different 3-hydroxypropionyl-CoA synthases in the 3-HP sub-pathway. M101: strain M1 harboring the plasmid pSC-HIS-*MsHPCS*-*MsACR*-*StHPCD* and pUGG-PH for overexpressing *MsHPCS*, *MsACR*, *StHPCD* and *MCR*; M102: M1 harboring the plasmid pSC-HIS-*SePrpE*-*MsACR*-*StHPCD* and pUGG-PH for overexpressing *SePrpE*, *MsACR*, *StHPCD* and *MCR*; M103, M102 with an additional copy of *SePrpE* expression cassette in XII-1 site. **B** The yield of the reporter mesaconate from glucose. M201, strain M2 harboring the plasmid pSC-HIS-*MCR* for overexpressing *MCR*; M203, strain M2 harboring the plasmid pUGG-PH for overexpressing *MCR*; M403, the *SePrpE* expression cassette replaced the *MsHPCS* expression cassette at the XI-2 site of M203, and an additional copy of the *SePrpE* expression cassette was integrated into the XII-1 site; M405, strain M403 with additional copy of *SePrpE* in the plasmid pUGG-PH. Strains were cultured with SC-dropout medium with 0.1% glyoxylate

To test the formation efficiency of 3-hydroxypropionyl-CoA from 3-HP, *Ms*HPCS, *Ms*ACR, *St*HPCD and *Ca*MCR were overexpressed in strain M1, resulting strain M101, and *Se*PrpE, *Ms*ACR, *St*HPCD and *Ca*MCR were overexpressed in strain M1, resulting strain M102. The production of mesaconate was used as an indicator compound for the optimization of 3-hydroxypropionyl-CoA synthase in the 3-HP sub-pathway. Compared to the expression of *Ms*HPCS in M101, the use of *Se*PrpE in M102 gave a much higher production of mesaconate, indicating a higher activity in the formation of 3-HP-CoA from 3-HP (Fig. 5A). To further improve the efficiency of the 3-HP sub-pathway, an additional copy of *SePrpE* was integrated into the XII-1 site (Jessop-Fabre et al. 2016) to obtain strain M103, resulting in a further 5.5-fold increase in the accumulation of mesaconate (Fig. 5A).

Finally, we constructed strain M403 and M405 by combining the above optimization strategies (balanced expression levels between MCR-C_mut_ and MCR-N, overexpression of MCR and PrpE). The yield of the reporter mesaconate of M403 (using *SePrpE* replaced *MsHPCS* at the XI-2 site of M203, and an additional copy of *SePrpE* was integrated into the XII-1 site) was increased to 6.62 mg/L (Fig. 5B), indicating the efficiency of the 3-HP sub-pathway has increased by 4.7 fold, compared with initial strain M201. Moreover, the production of mesaconate from glucose by the 3-HP sub-pathway in M403 is close to the strain M3 (Fig. 3), which produced around 5 mg/L mesaconate from the medium supplemented with 1 g/L of propionate and 1 g/L of glyoxylate. This highlights the MCL and MCH as limiting factors in the 3-HP sub-pathway. When comparing M403 to M405, it was found that an additional *SePrpE* gene expressed on the pUGG1 plasmid resulted in a further increase in mesaconate yield of up to 90.78 mg/L. This suggests that increasing the number of *SePrpE* copies can improve the efficiency of 3-HP and ultimately boost mesaconate production.

### Ethanol is more compatible with the 3-HP pathway in yeast

*S. cerevisiae* can utilize non-fermentable substrates such as ethanol, acetate, lactate, oleate and glycerol as carbon sources (Laxman et al. 2014; Lee et al. 2011; Li et al. 2020; Minard and McAlister-Henn 2005; Xiberras et al. 2019). Ethanol is an ordinary and inexpensive commodity substrate, which can be produced on an industrial scale from lignocellulosic biomass (Clomburg et al. 2017) and steel waste gas (Liew et al. 2016). During growth on ethanol, the glyoxylate cycle is critical (Daran-Lapujade et al. 2004) and can provide essential intermediate glyoxylate for the synthesis of mesconate via 3-HP pathway (Fig. 1). Meanwhile, ethanol can be used for the supply of the precursor acetyl-CoA without carbon release, and it does not belong to any of the intermediates in the 3-HP pathway. In the 3-HP pathway of mesocanate synthesis, using ethanol as the only carbon source, one molecule of mesocanate can fix one molecule of bicarbonate and two molecule ethanol. In contrast, when using glucose as the only carbon source, carbon fixation is not achieved, as pyruvate kinase catalyzes pyruvate to acetaldehyde, releasing one molecule of CO_2_ (bicarbonate). Therefore, the synthesis of one molecule of mesocanate requires two molecules of acetyl-CoA and releases one molecule of CO_2_ (bicarbonate) when glucose is used as the only carbon source. Those features make ethanol a suitable carbon source for the construction of 3-HP pathway in yeasts, and we attempted and optimized the 3-HP sub-pathway in *S. cerevisiae* using ethanol as the carbon source.

**Fig. 6:**
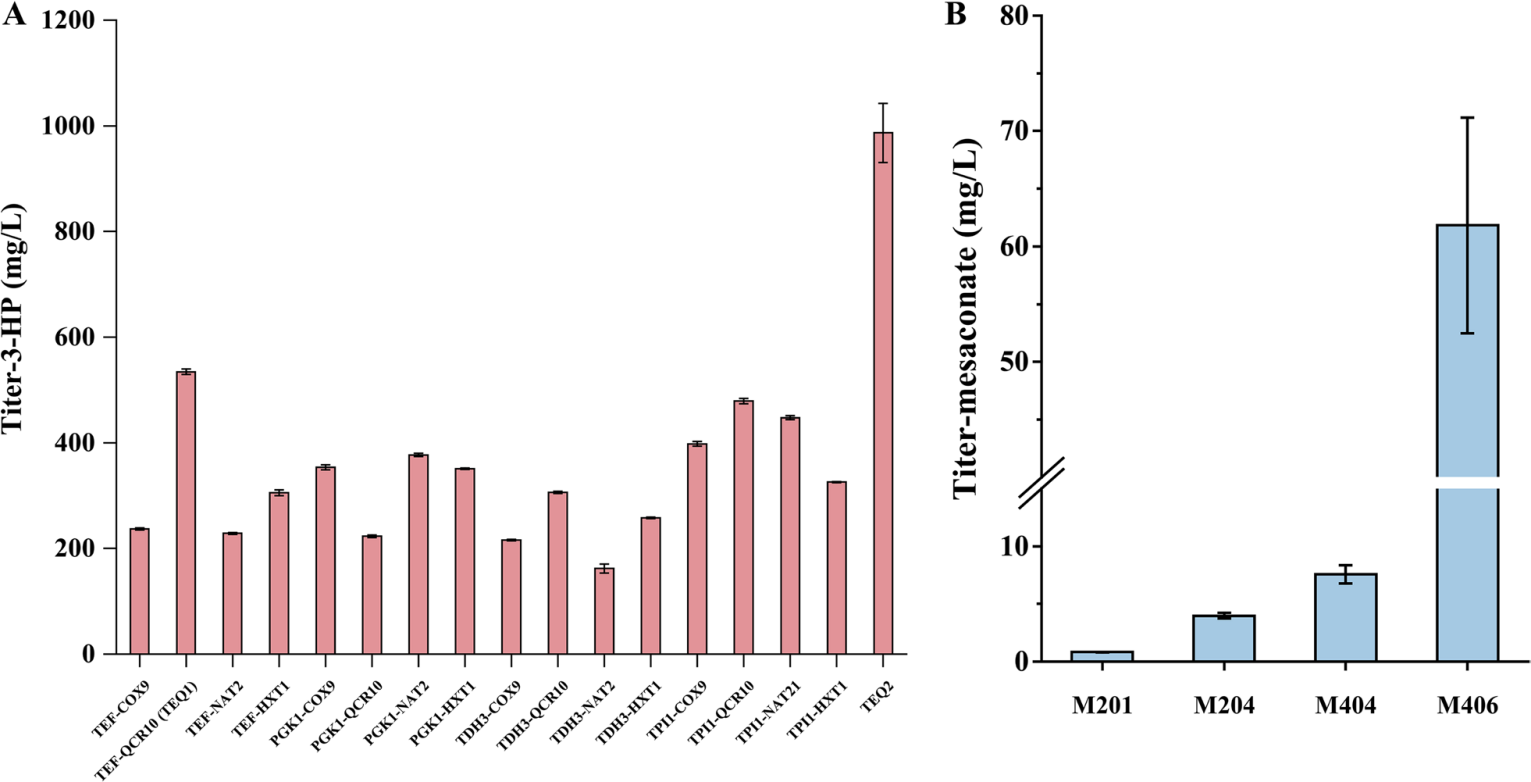
Optimization of the 3-HP sub-pathway in *S. cerevisiae* with ethanol as the carbon source. **A** 3-HP production in engineered yeast from ethanol by expression of MCR-C and MCR-N under control by different promoter combinations. **B** The production of the reporter mesaconate in engineered yeasts with ethanol as the carbon source. M201, strain M2 harboring the plasmid pSC-HIS-*MCR* for overexpressing *MCR*; M204, strain M2 harboring the plasmid pUGG-TEQ for overexpressing *MCR*; M404, the *SePrpE* expression cassette replaced the *MsHPCS* expression cassette at the XI-2 site of M204, and an additional copy of the *SePrpE* expression cassette was integrated into the XII-1 site; M406, strain M404 with additional copy of *SePrpE* in the plasmid pUGG-TEQ

Since the strength of the promoters varies under different carbon sources, we determined the strength of these promoters under ethanol by measuring the expression level of GFP. Similar to the results with glucose as the carbon source, the strength of the promoters *TEF1p*, *PGK1p*, *TDH3p* and *TPI1p* was higher than that of *QCR10p*, *COX9p*, *HXT1p* and *NAT2p* when ethanol was used as the carbon source (Additional file 1: Fig. S2). When glucose was used as the carbon source, the promoters used to drive the genes (*MsHPCS*/*SePrpE*, *StHPCD*, *MsACR*, *RsMCL* and *CaMCH*) of the 3-HP sub-pathway were *PGK1p*, *TEF1p*, *TDH3p*. So we didn't replace the promoters of these genes when the carbon source changed from glucose to ethanol. However, the relative strength between the promoters when ethanol was used as the carbon source (*PGK1p* ~ *TEF1p* ~ *TPI1p* > *TDH3p* > *QCR10p* > *COX9p* > *NAT2p* > *HXT1p*) was different compared to that when glucose was used as the carbon source (*PGK1p* ~ *TDH3p* > *TEF1p* ~ *TPI1p* > *QCR10p* > *HXT1p* > *COX9p* > *NAT2p*) (Additional file 1: Fig. S2). Then, we used *PGK1p*, *TEF1p*, *TDH3p*, *TPI1p* for the expression of MCR-C_mut_ and *HXT1p*, *QCR10p*, *COX9p*, *NAT1p* for the expression of MCR-N to balance the expression levels between two domains again when the carbon source changed from glucose to ethanol. As shown in Fig. 6A, a combination of *TEF1p*-MCR-C_mut_ and *QCR10p*-MCR-N produced the highest yield of 3-HP from ethanol. Furthermore, we used the previously adopted ultrahigh copy number plasmid pUGG to increase the overexpression of MCR, yield of 3-HP from ethanol increased to 987 mg/L (Fig. 6A).

To test the effect of optimization strategy of MCR on the 3-HP sub-pathway under ethanol condition, we transformed the ultrahigh copy number plasmid pUGG-TEQ into M2, obtaining strain M204. Similarly, the yield of the reporter mesaconate in M204 was increased to 3.97 mg/L, 3.78-fold higher than that of the initial strain M201 (0.83 mg/L from ethanol) (Fig. 6B). Next, we used *SePrpE* replaced *MsHPCS* at the XI-2 site of M204, and integrated an additional copy of *SePrpE* into the XII-1 site, obtaining strain M404. The reporter mesaconate yield derived from ethanol in M404 was augmented to 7.56 mg/L. M406, featuring supplementary SePrpE expression on pUGG1 plasmid, exhibited a mesaconate yield of 61.2 mg/L compared to M404.

The 3-HP pathway shows greater potentiality in CO_2_ fixation, and is considered to be the most suitable pathway for aerobic CO_2_ fixation (Liu et al. 2020). At present, studies related to the 3-HP cycle focused mainly on the production of 3-HP rather than C1 fixation (Yu et al. 2022). Here, we constructed part of the 3-HP pathway in recombinant *S. cerevisiae* and optimization of the recombinant strain significantly improved the efficiency of this pathway. Furthermore, the optimization of the 3-HP sub-pathway utilizing ethanol as a carbon source has shown the potential of using ethanol for this purpose. In the future, incorporating the engineered *S. cerevisiae* with the whole 3-HP pathway could enable the direct synthesis of biomass from CO_2_.

## Conclusion

Most of the intermediates in 3-HP pathway are acyl-CoA, which are difficult to quantify in cell extracts. Mesaconate is the corresponding organic acid of mesaconyl-C1-CoA, the intermediate metabolite in the 3-HP pathway. In this study, mesaconate was used as an indicator compound to assess the efficiency of reactions from acetyl-CoA to mesaconyl-C1-CoA, which facilitate the construction and optimization of the 3-HP sub-pathway in *S. cerevisiae* with the use of glucose or ethanol as the substrate. Currently, there are still some problems with the construction of the whole 3-HP pathway in yeast. The yeast's endogenous fatty acid synthesis pathway and acetyl-CoA hydrolases, like Ach1p, may compete with the 3-HP pathway for acetyl-CoA and mesaconyl-C1-CoA, respectively. The competition can lead to a shift in carbon metabolism, emphasizing mesaconate production over mesaconyl-C4-CoA in the process.

### Supplementary Information

**Additional file1: Fig. S1. **The 3-HP pathway, with the bicycle divided into four functional sub-pathways. Sub-pathway ①: Acetyl-CoA→Propionyl-CoA; Sub-pathway ②: Propionyl-CoA +Glyoxylate→Pyruvate+ Acetyl-CoA; Sub-pathway ③: Propionyl-CoA →Succinate-CoA; Sub-pathway ④: Succinate-CoA → Glyoxylate + Acetyl-CoA. Abbreviations: MCL, malyl-CoA/beta-methylmalyl-CoA/citramalyl-CoA lyase; MCH, 2-methylfumaryl-CoA hydratase; MCT, 2-methylfumaryl-CoA isomerase; MEH, 3-methylfumaryl-CoA hydratase. **Fig. S2. **A. Promoter optimization strategy for the expression of GFP under glucose or ethanol. The expression level of GFP under different promoters using glucoseor ethanolas carbon source. **Fig. S3. **Effects of adding 0.1% acetate on the growth of *Saccharomyces cerevisiae* with 2% ethanol. **Table S1. **List of plasmids used in this study. **Table S2.** List of strains used in this study.** Table S3. **List of primers used in this study. **Table S4.** Enzymatic properties of MCLs.

## Data Availability

The datasets used and/or analyzed during the current study are available from the corresponding author on reasonable request.
