# Supplementary material for: Exploiting a heterologous construction of the 3-hydroxypropionic acid carbon fixation pathway with mesaconate as an indicator in Saccharomyces cerevisiae
Source: Bioresour Bioprocess. 2023 May 24;10(1):33. doi: 10.1186/s40643-023-00652-5 (PMC10991142; doi:10.1186/s40643-023-00652-5)
Supplement: Supplementary file 1 — Additional file1: Fig. S1. The 3-HP pathway, with the bicycle divided into four functional sub-pathways. Sub-pathway ①: Acetyl-CoA→Propionyl-CoA; Sub-pathway ②: Propionyl-CoA +Glyoxylate→Pyruvate+ Acetyl-CoA; Sub-pathway ③: Propionyl-CoA →Succinate-CoA; Sub-pathway ④: Succinate-CoA → Glyoxylate + Acetyl-CoA. Abbreviations: MCL, malyl-CoA/beta-methylmalyl-CoA/citramalyl-CoA lyase; MCH, 2-methylfumaryl-CoA hydratase; MCT, 2-methylfumaryl-CoA isomerase; MEH, 3-methylfumaryl-CoA hydratase. Fig. S2. A. Promoter optimization strategy for the expression of GFP under glucose or ethanol. The expression level of GFP under different promoters using glucoseor ethanolas carbon source. Fig. S3. Effects of adding 0.1% acetate on the growth of Saccharomyces cerevisiae with 2% ethanol. Table S1. List of plasmids used in this study. Table S2. List of strains used in this study. Table S3. List of primers used in this study. Table S4. Enzymatic properties of MCLs. [file 40643_2023_652_MOESM1_ESM.docx]

*Additional files*

**Exploiting a heterologous construction of the 3-hydroxypropionic acid carbon fixation pathway with mesaconate as an indicator in *Saccharomyces cerevisiae***

Shijie Xu^1 &^, Weibo Qiao^1 &^, Zuanwen Wang^1^, Xiaoying Fu^1^, Zihe Liu^1^ and Shuobo Shi^1^ **^*^**

^1^Beijing Advanced Innovation Center for Soft Matter Science and Engineering, College of Life Science and Technology, Beijing University of Chemical Technology, Beijing 100029, China

**Author Contributions:**

^&^ Weibo Qiao and Shijie Xu contributed equally.

**Correspondence:**

*E-mail: shishuobo@mail.buct.edu.cn


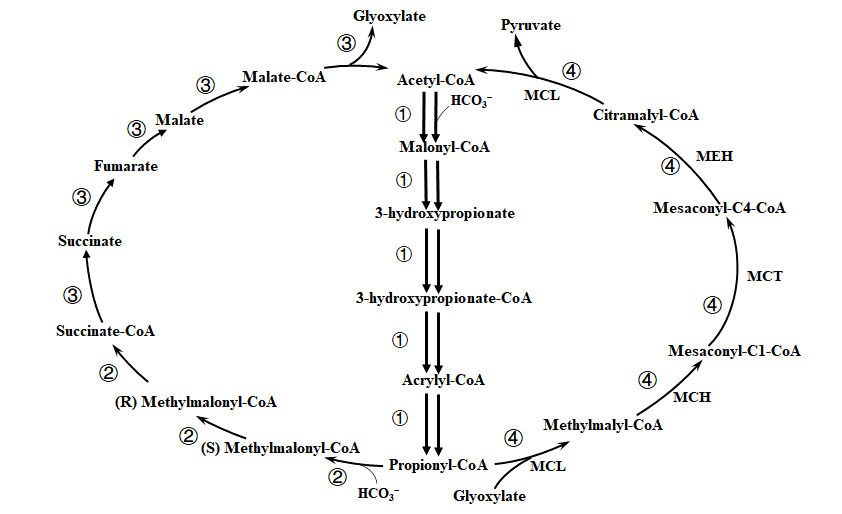


**Fig. S1** The 3-HP pathway, with the bicycle divided into four functional sub-pathways (redrawn from ^[1]^). Sub-pathway ①: Acetyl-CoA→Propionyl-CoA; Sub-pathway ②: Propionyl-CoA +Glyoxylate→Pyruvate+ Acetyl-CoA; Sub-pathway ③: Propionyl-CoA →Succinate-CoA; Sub-pathway ④: Succinate-CoA → Glyoxylate + Acetyl-CoA. Abbreviations: MCL, malyl-CoA/beta-methylmalyl-CoA/citramalyl-CoA lyase; MCH, 2-methylfumaryl-CoA hydratase; MCT, 2-methylfumaryl-CoA isomerase; MEH, 3-methylfumaryl-CoA hydratase.

**
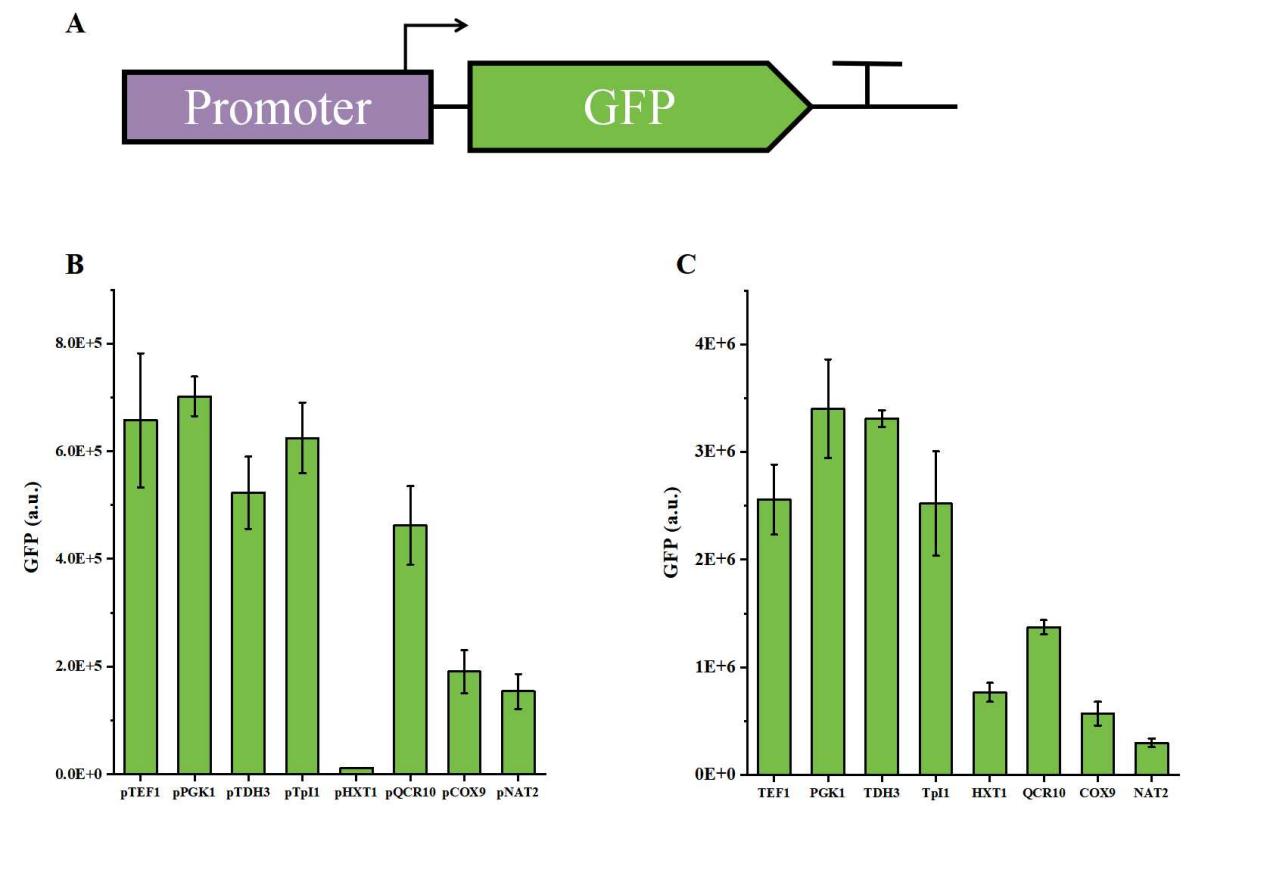
**

**Fig. S2** A. Promoter optimization strategy for the expression of GFP under glucose or ethanol. The expression level of GFP under different promoters using glucose (B) or ethanol (C) as carbon source.

**
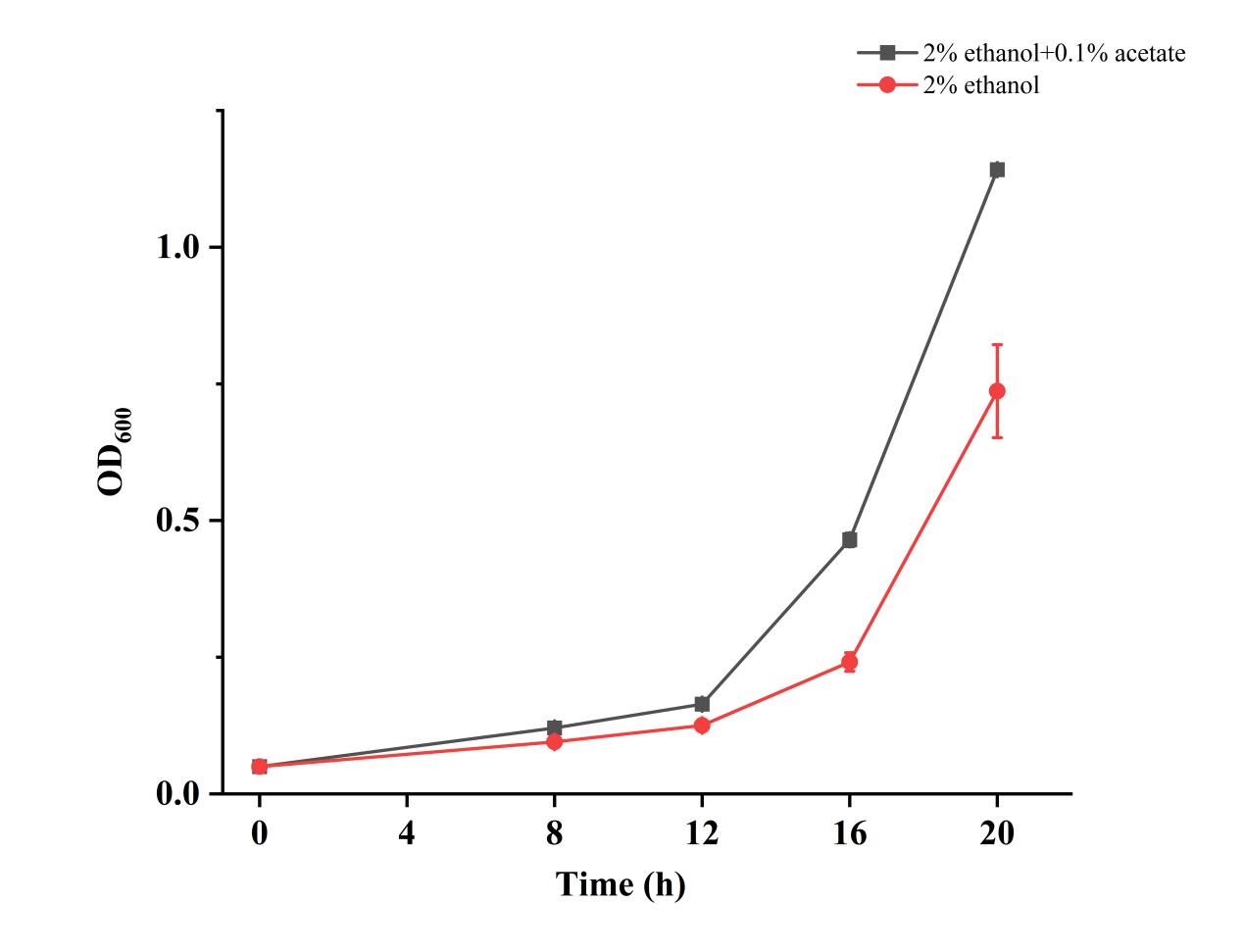
**

**Fig. S3** Effects of adding 0.1% acetate on the growth of *Saccharomyces cerevisiae* with 2% ethanol

**Table S1.** List of plasmids used in this study.

| **Plasmid** | **Description** | **Source** |
| --- | --- | --- |
| pESC-HIS | *Amp^r^, HIS1, 2 μ ori,* P*_GAL1_-*T*_CYC1_,* P*_GAL10_-*T*_ADH1_* | Thermo Fisher |
| pESC-TRP | *Amp^r^, TRP1, 2 μ ori,* P*_GAL1_-*T*_CYC1_,* P*_GAL10_-*T*_ADH1_* | Thermo Fisher |
| pESC-URA | *Amp^r^, URA3, 2 μ ori,* P*_GAL1_-*T*_CYC1_,* P*_GAL10_-*T*_ADH1_* | Thermo Fisher |
| pYX212 | *Amp^r^, URA3, 2 μ ori,*TPI promoter | R & D Systems |
| pUGG1 | Amp*^r^, URA3* with truncated promoter*, 2 μ ori;* ultrahigh copy number plasmid | ^[2]^ |
| pET-28a | Expression plasmid; Km^r^ | Stratagene |
| pCas | Amp*^r^*, P*_TEF1_*-*iCas9*-T*_ADH1_*, P*_SNR52_*; Cas9 CRISPR vector for sequence-specific yeast genome integration | ^[3]^ |
| pScURA3 | PCR template for gRNA-URA3-SNR52p-tGly | ^[3]^ |
| pSC-HIS | pESC-HIS, P*_TEF1_-*T*_CYC1_,* P*_PGK1_-*T*_ADH1_* (replace P*_GAL1_-*T*_CYC1_,* P*_GAL10_-*T*_ADH1_*) | This study |
| pSC-TRP | pESC-TRP, P_T_*_EF1_-*T*_CYC1_,* P*_PGK1_-*T*_ADH1_* (replace P*_GAL1_-*T*_CYC1_,* P*_GAL10_-*T*_ADH1_*) | This study |
| pSC-URA | pESC-URA, P*_TEF1_-*T*_CYC1_,* P*_PGK1_-*T*_ADH1_* (replace P*_GAL1_-*T*_CYC1_,* P*_GAL10_-*T*_ADH1_*) | This study |
| pSC-HIS-*SePrpE*-*RsMCL* | pSC-HIS, P*_TEF1_-SePrpE-*T*_CYC1_,* P*_PGK1_-RsMCL-*T*_ADH1_* | This study |
| pSC-TRP-*YciA* | pSC-TRP, P*_TEF1_-YciA-*T*_CYC1_* | This study |
| pSC-TRP-*ACH1* | pSC-TRP, P*_TEF1_-ACH1-*T*_CYC1_* | This study |
| pSC-TRP-*EHD3* | pSC-TRP, P*_TEF1_-EHD3-*T*_CYC1_* | This study |
| pSC-TRP-*TES1* | pSC-TRP, P*_TEF1_-TES1-*T*_CYC1_* | This study |
| pYX212-*CaMCH* | pYX212 with *CaMCH* | This study |
| pYX212-*RsMCH* | pYX212 with *RsMCH* | This study |
| pYX212-*HmMCH* | pYX212 with *HmMCH* | This study |
| pSC-URA-P*_TEF1_*-*GFP* | pSC-URA, P*_TEF1_*-*GFP-*T*_ADH1_* | This study |
| pSC-URA-P*_PGK1_*-*GFP* | pSC-URA, P*_PGK1_*-*GFP-*T*_ADH1_* | This study |
| pSC-URA-P*_TPI1_*-*GFP* | pSC-URA, P*_TPI1_*-*GFP-*T*_ADH1_* | This study |
| pSC-URA-P*_TDH3_*-*GFP* | pSC-URA, P*_TDH3_*-*GFP-*T*_ADH1_* | This study |
| pSC-URA-P*_COX9_*-*GFP* | pSC-URA, P*_COX9_*-*GFP-*T*_ADH1_* | This study |
| pSC-URA-P*_NAT2_*-*GFP* | pSC-URA, P*_NAT2_*-*GFP-*T*_ADH1_* | This study |
| pSC-URA-P*_QCR10_*-*GFP* | pSC-URA, P*_QCR10_*-*GFP-*T*_ADH1_* | This study |
| pSC-URA-P*_HXT1_*-*GFP* | pSC-URA, P*_HXT1_*-*GFP-*T*_ADH1_* | This study |
| pSC-HIS-*MCR* | pSC-HIS-P*_TEF1_*-*MCR*_mut_-T*_CYC1_*; MCR_mut_, mutation in N940V, K1106W and S1114R | This study |
| pSC-HIS-*MCR*-*YciA* | pSC-HIS-P*_TEF1_*-*MCR*_mut_-T*_CYC1_*_,_ P*_PGK1_*-*YciA*_t_-T*_ADH1_* | This study |
| pSC-HIS-TDH | pSC-HIS-P*_TDH3_*-*MCR*-C_mut_-T*_ADH1_*, P*_HXT1_*-*MCR*-N-T*_CYC1_* | This study |
| pSC-HIS-TDC | pSC-HIS, P*_TDH3_*-*MCR*-C_mut_-T*_ADH1_*, P*_COX9_*-*MCR*-N-T*_CYC1_* | This study |
| pSC-HIS-TQ | pSC-HIS, P*_TDH3_*-*MCR*-C_mut_-T*_ADH1_*, P*_QCR10_*-*MCR*-N-T*_CYC1_* | This study |
| pSC-HIS-TDN | pSC-HIS, P*_TDH3_*-*MCR*-C_mut_-T*_ADH1_*, P*_NAT2_*-*MCR*-N-T*_CYC1_* | This study |
| pSC-HIS-PH | pSC-HIS, P*_PGK1_*-*MCR*-C_mut_-T*_ADH1_*, P*_HXT1_*-*MCR*-N-T*_CYC1_* | This study |
| pSC-HIS-PC | pSC-HIS, P*_PGK1_*-*MCR*-C_mut_-T*_ADH1_*, P*_COX9_*-*MCR*-N-T*_CYC1_* | This study |
| pSC-HIS-PQ | pSC-HIS, P*_PGK1_*-*MCR*-C_mut_-T*_ADH1_*, P*_QCR10_*-*MCR*-N-T*_CYC1_* | This study |
| pSC-HIS-PN | pSC-HIS, P*_PGK1_*-*MCR*-C_mut_-T*_ADH1_*, P*_NAT2_*-*MCR*-N-T*_CYC1_* | This study |
| pSC-HIS-TPH | pSC-HIS, P*_TPI1_*-*MCR*-C_mut_-T*_ADH1_*, P*_HXT1_*-*MCR*-N-T*_CYC1_* | This study |
| pSC-HIS-TPC | pSC-HIS, P*_TPI1_*-*MCR*-C_mut_-T*_ADH1_*, P*_COX9_*-*MCR*-N-T*_CYC1_* | This study |
| pSC-HIS-TPQ | pSC-HIS, P*_TPI1_*-*MCR*-C_mut_-T*_ADH1_*, P*_QCR10_*-*MCR*-N-T*_CYC1_* | This study |
| pSC-HIS-TPN | pSC-HIS, P*_TPI1_*-*MCR*-C_mut_-T*_ADH1_*, P*_NAT2_*-*MCR*-N-T*_CYC1_* | This study |
| pSC-HIS-TEH | pSC-HIS, P*_TEF1_*-*MCR*-C_mut_-T*_ADH1_*, P*_HXT1_*-*MCR*-N-T*_CYC1_* | This study |
| pSC-HIS-TEC | pSC-HIS, P*_TEF1_*-*MCR*-C_mut_-T*_ADH1_*, P*_COX9_*-*MCR*-N-T*_CYC1_* | This study |
| pSC-HIS-TEQ | pSC-HIS, P*_TEF1_*-*MCR*-C_mut_-T*_ADH1_*, P*_QCR10_*-*MCR*-N-T*_CYC1_* | This study |
| pSC-HIS-TEN | pSC-HIS, P*_TEF1_*-*MCR*-C_mut_-T*_ADH1_*, P*_NAT2_*-*MCR*-N-T*_CYC1_* | This study |
| pSC-URA-PH | pSC-URA, P*_PGK1_*-*MCR*-C_mut_-T*_ADH1_*, P*_HXT1_*-*MCR*-N-T*_CYC1_* | This study |
| pSC-URA-TEQ | pSC-URA, P*_TEF1_*-*MCR*-C_mut_-T*_ADH1_*, P*_QCR10_*-*MCR*-N-T*_CYC1_* | This study |
| pUGG-PH | pUGG1, P*_PGK1_*-*MCR*-C_mut_-T*_ADH1_*, P*_HXT1_*-*MCR*-N-T*_CYC1_* | This study |
| pUGG-PH-*SePrpE* | pUGG1, P*_PGK1_*-*MCR*-C_mut_-T*_ADH1_*, P*_HXT1_*-*MCR*-N-T*_CYC1,_* P*_FBA1_-SePrpE-*T*_RPS2_* | This study |
| pUGG-TEQ | pUGG1, P*_TEF1_*-*MCR*-C_mut_-T*_ADH1_*, P*_QCR10_*-*MCR*-N-T*_CYC1_* | This study |
| pUGG-TEQ-*SePrpE* | pUGG1, P*_TEF1_*-*MCR*-C_mut_-T*_ADH1_*, P*_QCR10_*-*MCR*-N-T*_CYC1,_* P*_FBA1_-SePrpE-*T*_RPS2_* | This study |
| pSC-HIS-*MsHPCS*-*MsACR*-*StHPCD* | pSC-HIS, P*_TEF1_*-*MsHPCS*-T*_ADH2_*, P*_PGK1_*-*StHPCD*-T*_CYC1_*, P*_TDH3_*-*MsACR*-T*_PYK1_*, | This study |
| pSC-HIS-*SePrpE*-*MsACR*-*StHPCD* | pSC-HIS, P*_TEF1_*-*SePrpE*-T*_ADH2_*, P*_PGK1_*-*StHPCD*-T*_CYC1_*, P*_TDH3_*-*MsACR*-T*_PYK1_*, | This study |
| pCas-*ΔACH1* | pCas, P*_SNR52_*-gRNA*_ACH1_*-T*_SNR52_*; Cas9 CRISPR vector for knockout of *ACH1* | This study |
| pCas-*ΔEHD3* | pCas, P*_SNR52_*-gRNA*_EHD3_*-T*_SNR52_*; Cas9 CRISPR vector for knockout of *EHD3* | This study |
| pCas-*ΔTES1* | pCas, P*_SNR52_*-gRNA*_TES1_*-T*_SNR52_*; Cas9 CRISPR vector for knockout of *TES1* | This study |
| pCas-XII-1 | Cas9 CRISPR vector for integration at XII-1^[4]^ site | Stored in the Shi lab |
| pCas-XI-2 | Cas9 CRISPR vector for integration at XI-2^[4]^ site | Stored in the Shi lab |
| pCas-XI-3 | Cas9 CRISPR vector for integration at XI-3^[4]^site | Stored in the Shi lab |

**Table S2.** List of strains used in this study.

| **Name** | **Genotype** | **Source** |
| --- | --- | --- |
| *CEN.PK 2-1D* | *MATα; ura3-52; trp1-289; leu2-3,112; his3Δ 1; MAL2-8C; SUC2* | *EUROSCARF* |
| TEF1p | *CEN.PK 2-1D,* pSC-URA-P*_TEF1_*-*GFP* | This study |
| PGK1p | *CEN.PK 2-1D,* pSC-URA-P*_PGK1_*-*GFP* | This study |
| TPI1p | *CEN.PK 2-1D,* pSC-URA-P*_TPI1_*-*GFP* | This study |
| TDH3p | *CEN.PK 2-1D,* pSC-URA-P*_TDH3_*-*GFP* | This study |
| COX9p | *CEN.PK 2-1D,* pSC-URA-P*_COX9_*-*GFP* | This study |
| NAT2p | *CEN.PK 2-1D,* pSC-URA-P*_NAT2_*-*GFP* | This study |
| QCR10p | *CEN.PK 2-1D,* pSC-URA-P*_QCR10_*-*GFP* | This study |
| HXT1p | *CEN.PK 2-1D,* pSC-URA-P*_HXT1_*-*GFP* | This study |
| TDH3-HXT1 | *CEN.PK 2-1D,* pSC-HIS-TDH | This study |
| TDH3-COX9 | *CEN.PK 2-1D,* pSC-HIS-TDC | This study |
| TDH3-QCR10 | *CEN.PK 2-1D,* pSC-HIS-TQ | This study |
| TDH3-NAT2 | *CEN.PK 2-1D,* pSC-HIS-TDN | This study |
| PGK1-HXT1 (PH1) | *CEN.PK 2-1D,* pSC-HIS-PH | This study |
| PGK1-COX9 | *CEN.PK 2-1D,* pSC-HIS-PC | This study |
| PGK1-QCR10 | *CEN.PK 2-1D,* pSC-HIS-PQ | This study |
| PGK1-NAT2 | *CEN.PK 2-1D,* pSC-HIS-PN | This study |
| TPI1-HXT1 | *CEN.PK 2-1D,* pSC-HIS-TPH | This study |
| TPI1-COX9 | *CEN.PK 2-1D,* pSC-HIS-TPC | This study |
| TPI1-QCR10 | *CEN.PK 2-1D,* pSC-HIS-TPQ | This study |
| TPI1-NAT2 | *CEN.PK 2-1D,* pSC-HIS-TPN | This study |
| TEF1-HXT1 | *CEN.PK 2-1D,* pSC-HIS-TEH | This study |
| TEF1-COX9 | *CEN.PK 2-1D,* pSC-HIS-TEC | This study |
| TEF1-QCR10 (TEQ1) | *CEN.PK 2-1D,* pSC-HIS-TEQ | This study |
| TEF1-NAT2 | *CEN.PK 2-1D,* pSC-HIS-TEN | This study |
| PH2 | *CEN.PK 2-1D,* pSC-URA-PH | This study |
| PH3 | *CEN.PK 2-1D,* pUGG-PH | This study |
| TEQ2 | *CEN.PK 2-1D,* pUGG-TEQ | This study |
| M0 | *CEN.PK 2-1D*, pSC-HIS-*SePrpE*-*RsMCL* | This study |
| M0-C | M0, pYX212 | This study |
| M0-*CaMCH* | M0, pYX212-*CaMCH* | This study |
| M01 | M0, pSC-TRP-*YciA* | This study |
| M01-C | M01, pYX212 | This study |
| M01-*CaMCH* | M01, pYX212-*CaMCH* | This study |
| M01-*RsMCH* | M01, pYX212-*RsMCH* | This study |
| M01-*HmMCH* | M01, pYX212-*HmMCH* | This study |
| M1 | CEN.PK 2-1D*,* *XI-3::P_TDH3_-RsMCL-T_PYK1_, P_TEF1_-CaMCH-T_CYC1_* | This study |
| M101 | M1, pSC-HIS-*MsHPCS*- *MsACR*-*StHPCD*, pUGG-PH | This study |
| M102 | M1, pSC-HIS-*SePrpE*- *MsACR*-*StHPCD*, pUGG-PH | This study |
| M103 | M102, *XII-1::P_PGK1_-SePrpE-T_CYC1_* | This study |
| M2 | M1, *XI-2::*P*_TEF1_*-*MsHPCS*-T*_ADH2_*, P*_PGK1_*-*StHPCD*-T*_CYC1_*, P*_TDH3_*-*MsACR*-T*_PYK1_* | This study |
| M201 | M2, pSC-HIS-*MCR* | This study |
| M202 | M2, pSC-HIS-*MCR*-*YciA* | This study |
| M203 | M2, pUGG-PH | This study |
| M204 | M2, pUGG-TEQ | This study |
| M3 | M1, *XII-1::P_PGK1_-SePrpE-T_CYC1_* | This study |
| M3-TRP | M3, pSC-TRP | This study |
| M3-*YciA* | M3, pSC-TRP-*YciA* | This study |
| M3-*ACH1* | M3, pSC-TRP-*ACH1* | This study |
| M3-*EHD3* | M3, pSC-TRP-*EHD3* | This study |
| M3-*TES1* | M3, pSC-TRP-*TES1* | This study |
| M3-*ΔACH1* | M3, *ach1Δ* | This study |
| M3-*ΔEHD3* | M3, *ehd3Δ* | This study |
| M3-*ΔTES1* | M3, *tes1Δ* | This study |
| M4 | M3, *XI-2::P_TEF1_-SePrpE-T_ADH2_,* P*_PGK1_*-*StHPCD*-T*_CYC1_*, P*_TDH3_*-*MsACR*-T*_PYK1_* | This study |
| M403 | M4, pUGG-PH | This study |
| M404 | M4, pUGG-TEQ | This study |
| M405 | M4, pUGG-PH-*SePrpE* | This study |
| M406 | M4, pUGG-TEQ-*SePrpE* | This study |

**Table S3.** List of primers used in this study.

| **Primer name** | **Primer Sequence (5'-3')** | **Description of the PCR product** |
| --- | --- | --- |
| psc-PGK1p-EcoRI-f | tttagtgagggttgaattcttgttttatatttgttgtaa | Construction of pSC-URA, pSC-HIS, pSC-TRP plasmid |
| psc-TEF1p-BamHI-r | gtgagtcgtattacggatccgttgtaattaaaacttagat |  |
| psc-prpe-f | ctaagttttaattacaacggatccATGTCCTTCTCCGAATTTTA | Construction of pSC-HIS-*SePrpE*-*RsMCL* plasmid |
| psc-prpe-r | gccctatagtgagtcgtattacTTATTCCTCGATGGCCTGTCT |  |
| psc-rsmcl-f | caacaaatataaaacaagaattcAtgagcttccgccttcagc |  |
| psc-rsmcl-r | CTtgtaatccatcgatactagggccgagatcatttctGCC |  |
| MCR-C-PCSh-R | agaattgttaattaagagctcTTAGACTGTAATGGCTCTACCTCTGTG | Construction of pSC-HIS-*MCR* plasmid |
| MCR-K1106W-S1114R-F | gatggattgctttgtctgatggtgctagattagcattggtaaccccagaaac |  |
| MCR-K1106W-S1114R-R | tctagcaccatcagacaaagcaatccatctggcgactctgaagtga |  |
| MCR-N940V-R | caccggaaacaactctgtcagctaagtaatagacag |  |
| MCR-N940V-F | cttagctgacagagttgtttccggtgaaacttttca |  |
| MCR-C-PCSh-F | tagggcccgggcgtcgacGCGGCCGCACTAGTATGTCCGCTACCACTGGTG |  |
| M,N,C-CK-F | acttcaggttgtctaactcc |  |
| M,N,C-CK-R | aaatttctggcaaggtagac |  |
| MCR-N-PCSh-R | acatactagtgcggccgcGTCGACGCCCGGGCCCTATGAGTGGTACAGGTAGATTAGC |  |
| MCR-N-PCSh-F | gcggtaccaagcttactcgagTTAGATGTTGGCTGGTATGTTCAAT |  |
| L-TPI-F | gCACCAGTGGTAGCGGACATACTAGTtgtatgtgttttttgtagttatagatttaagc | Construction of pSC-HIS-TDH, pSC-HIS-TDC, pSC-HIS-TQ,  pSC-HIS-TDN,  pSC-HIS-PH,  pSC-HIS-PC,  pSC-HIS-PQ,  pSC-HIS-PN,  pSC-HIS-TPH,  pSC-HIS-TPC,  pSC-HIS-TPQ,  pSC-HIS-TPN,  pSC-HIS-TEH,  pSC-HIS-TEC,  pSC-HIS-TEQ,  pSC-HIS-TEN plasmid |
| L-TPI-R | GTAGGTCGATGGATCCATGCTGCAGgatctacgtatggtcattcttcttc |  |
| L-HXT1-F | CTGCAGCATGGATCCATCGACCTACGGCCACAATGAAACTTCAATTC |  |
| L-HXT1-R | CTAATCTACCTGTACCACTCATAGGGCCGATTTTACGTATATCAACTAGTTGACG |  |
| L-TDH3-F | gCACCAGTGGTAGCGGACATACTAGTTTTGTTTGTTTATGTGTGTTTATTCG |  |
| L-TDH3-R | GTAGGTCGATGGATCCATGCTGCAGATACTAGCGTTGAATGTTAGCG |  |
| L-COX9-F | CTGCAGCATGGATCCATCGACCTACGCTGGGCGATCTTCC |  |
| L-COX9-R | CTAATCTACCTGTACCACTCATAGGGCCGTCTGTGTAAGTCGC |  |
| L-TEF1-F | gCACCAGTGGTAGCGGACATACTAGTttgtaattaaaacttagattagattgctatgc |  |
| L-TEF1-R | GTAGGTCGATGGATCCATGCTGCAGgcacacaccatagcttcaaaa |  |
| L-PGK1-F | gCACCAGTGGTAGCGGACATACTAGTttgttttatatttgttgtaaaaagtagataattac |  |
| L-PGK1-R | GTAGGTCGATGGATCCATGCTGCAGggaagtaccttcaaagaatgg |  |
| L-NAT2-F | CTGCAGCATGGATCCATCGACCTACGATAATTAACTTTTGTTAGTTTTTTATTAATAATATATCATTACAAG |  |
| L-NAT2-R | CTAATCTACCTGTACCACTCATAGGGCCCACCCCAACTCTAGACCAAAAAAG |  |
| L-QCR-F | CTGCAGCATGGATCCATCGACCTACGCTTCTCATTCTATTTTAATTATACTAGTACG |  |
| L-QCR-R | CTAATCTACCTGTACCACTCATAGGGCCTGAGGTTAGTTTTAAGTCTTATGGATTG |  |
| GFP-Xhol1-F | gcggtaccaagcttactcgagatgtctaaaggtgaagaattattcac | Construction of pSC-URA-P*_COX9_*-*GFP* plasmid |
| GFP-Bcu1-R | ccttgtaatccatcgatactagtttaTAGCTTGGAtttgtacaattcatcc |  |
| ADH-CK-R | CCTGAGAAAGCAACCTGAC |  |
| CYC-CK-F | CAAAACCTTCTCAAGCAAGG |  |
| GFP-COX9-F | ttagagcggatcttagctagcGCTGGGCGATCTTCC |  |
| GFP-COX9-R | CttcacctttagacatctcgagGTCTGTGTAAGTCGCTTGTAGTTAG |  |
| GFP-TDH3-F | ttagagcggatcttagctagcagtttatcattatcaatactcgcc | Construction of pSC-URA-P*_TDH3_*-GFP plasmid |
| GFP-TDH3-R | Cttcacctttagacatctcgagatccgtcg |  |
| GFP-HXT1-F | ttagagcggatcttagctagcGGCCACAATGAAACTTCAATTC | Construction of pSC-URA-P*_HXT1_*-GFP plasmid |
| GFP-HXT1-R | CttcacctttagacatctcgagGATTTTACGTATATCAACTAGTTGACG |  |
| GFP-NAT2-F | ttagagcggatcttagctagcGATAATTAACTTTTGTTAGTTTTTTATTAATAATATATCATTAC | Construction of pSC-URA-P*_NAT2_*-*GFP* plasmid |
| GFP-NAT2-R | CttcacctttagacatctcgagCACCCCAACTCTAGACCAAA |  |
| GFP-QCR10-F | ttagagcggatcttagctagcGCTTCTCATTCTATTTTAATTATACTAGTAC | Construction of pSC-URA-P*_QCR10_*-*GFP* plasmid |
| GFP-QCR10-R | CttcacctttagacatctcgagTGAGGTTAGTTTTAAGTCTTATGGATTG |  |
| GFP-TPI-F | ttagagcggatcttagctagcGATCTACGTATGGTCATTCTTCTTC | Construction of pSC-URA-P*_TPI1_*-*GFP* plasmid |
| GFP-TPI-R | CttcacctttagacatctcgagTGTATGTGTTTTTTGTAGTTATAGATTTAAGC |  |
| GFP-TEF1-F | ttagagcggatcttagctagcGCACACACCATAGCTTCAAA | Construction of pSC-URA-P*_TEF1_*-*GFP* plasmid |
| GFP-TEF1-R | CttcacctttagacatctcgagTTGTAATTAAAACTTAGATTAGATTGCTATGC |  |
| GFP-PGK1-F | ttagagcggatcttagctagcGGAAGTACCTTCAAAGAATGGG | Construction of pSC-URA-P*_PGK1_*-*GFP* plasmid |
| GFP-PGK-R | ttcacctttagacatctcgagTTGTTTTATATTTGTTGTAAAAAGTAGATAATTACTTC |  |
| TEQ-Sal1-R | gcacttttcggAACGGTCGACttcgatcatatgcttcgagcgtcccaaaac | Construction of pSC-URA-TEQ plasimd |
| TEQ-Bgl2-F | TTTACGGTTCCTGGCCAGATCTgagcgacctcatgctatacc |  |
| ycia-Nde1-F | ttgggacgctcgaagcatatggcacacaccatagcttcaaaatg | Construction of pSC-TRP-*YciA* plasmid |
| ycia-Sal1-R | gcacttttcggAACGGTCGACcttcgagcgtcccaaaac |  |
| Ura-F | ctgaagttcctatactttctagagaat | Construction of pSC-URA-PH, pUGG-PH, pUGG-TEQ plasmid |
| Ura-CK-F | catataaggaacgtgctgc |  |
| Ura-CK-R | gccgcatcttctcaaatatg |  |
| Ura-R | GCCATTACAGTCTAAgagctcttaattaacaattcttcgc |  |
| MCR-PH-Bgl2-F | TTACGGTTCCTGGCCAGATCTTgagcgacctcatgctatacc |  |
| MCR-PH-Sal1-R | gcacttttcggAACGGTCGACGCGAAGAGcttcgagcgtcccaaaacc |  |
| FBA-PH-sal1-F | ctcgaagCTCTTCGCGTCGACGGTGTGTGCATAACAATACTG | Construction of pUGG-PH-*SePrpE* plasimd |
| FBA-PH-sal1-R | gcacttttcggAACGGTCGACAACGCCCcTAAAATTTTGATC |  |
| FBA-TEQ-sal1-F | cgaagcatatgatcgaaGTCGACGGTGTGTGCATAACAATACTG | Construction of pUGG-TEQ-*SePrpE* plasimd |
| FB1-TEQ-sal1-R | ggcacttttcggAACGGTCGACAACGCCCcTAAAATTTTGATC |  |
| OVER-CSDR-F | GGGTACCGGGCCCCC | Construction of pSC-HIS-*MsHPCS*- *MsACR*-*StHPCD* plasmid |
| CS-F | GGGTACCGGGCCCCCCCTCGAGGTCGACaatgctactattttggagattaatct |  |
| CS-R | gcagatgttATAATATCTGTGCGTAGCTACTAATAGGATAAATTATAGGAATTtataac |  |
| CD-F | ACGCACAGATATTATaacatctgc |  |
| CD-R | CCGCAAATTAAAGCCTTCG |  |
| ACR-F | CGAAGGCTTTAATTTGCGGaaacagtttatcattatcaatactcgcc |  |
| ACR-R | TGGCGGCCGCTCTAGAACTAGTGGATCCcatttatgtacccatgtataacctt |  |
| OVER-CSDR-R | TGGCGGCCGCTCTAGAAC |  |
| prpe-3hp-Apa1-F | GGCGAATTGGGTACCGGGCCCgcacacaccatagcttcaaaatg | Construction of pSC-HIS-*SePrpE*- *MsACR*-*StHPCD* plasmid |
| prpe-3hp-Nco1-R | aaccgggtccgtctaccatggTTATTCCTCGATGGCCTGTCTG |  |
| XI2-TEF-F | ttccacaagtaaacccttaagGCACACACCATAGCTTCAAAATG | Insertion of gene P*_TEF1_*-*MsHPCS*-T*_ADH2_*, P*_PGK1_*-*StHPCD*-T*_CYC1_*, P*_TDH3_*-*MsACR*-T*_PYK1_* and *P_TEF1_-SePrpE-T_ADH2_,* P*_PGK1_*-*StHPCD*-T*_CYC1_*, P*_TDH3_*-*MsACR*-T*_PYK1_* at XI2 site |
| XI2-PYK1t-R | ctagtcgtgtgtacccttaagCATTTATGTACCCATGTATAACCTTCC |  |
| XI2-CK-F | GGAAAGTCGCCACTCATC |  |
| XI2-CK-R | TAGCCACGAAAACTGCAAAG |  |
| Prpe-XII1-F1 | AAATTTTTTTGGCAAATCCCAGATTTGGCTTTGATTTTGGCATCGGTTCGGTTCgcacacaccatagcttcaaaat | Insertion of gene *P_PGK1_-SePrpE-T_CYC1_* at XII2 site |
| Prpe-XII1-F2 | TAATTTTCCTCGCCTTTCATATTTCGTATCTTTATTCTATATCCTAAAATTTTTTTGGCAAATCCCAG |  |
| Prpe-XII1-R1 | TTACAGTCACTCAGACAGAGCACTAAACTGAACTAGTTATTAAGGTATGTGCAGTTGcttcgagcgtcccaaaac |  |
| Prpe-XII1-R2 | CTTACCAATATATGTCATGAGAAGTGGCTTGTAAAATTTATTATTACAGTCACTCAGACAGAGC |  |
| ACH1-F | gttttaattacaacccggatccATGACAATTTCTAATTTGTTAAAGCAG | Construction of pSC-TRP-*ACH1* plasmid |
| ACH1-R | caacttctgttccatGTCAACTGGTTCCCAGCTGTCG |  |
| TES1-f | ctaatctaagttttaattacaacccggatccATGAGTGCTTCCAAAATGGCC | Construction of pSC-TRP-*TES1* plasmid |
| TES1-r | gaaatcaacttctgttccatgtcgacGAACTTGGCTCGAATGTCTCGT |  |
| EHD3-f： | ctaatctaagttttaattacaacccggatccATGCTCAGAAATACGCTAAAATGTG | Construction of pSC-TRP-*EHD3* plasmid |
| EHD3-r： | cggaaatcaacttctgttccatatgTTTCCATCTTAAGCCATCGTTAAC |  |
| pSC-YciA-BamHI-f | ctaatctaagttttaattacaacccggatccatgtctacaacacataacgtccctc | Construction of pSC-HIS-*MCR*-*YciA* plasmid |
| pSC-YciA-SalI-r | gaaatcaacttctgttccatgtcgacctcaacgggtaaggcgcgag |  |
| gACH1-f | aaaGGTCTCAGATCGTCCCAGGTCCCTCTGTCGGGTTTTAGAGCTAGAAATAGCAAGTTA | Construction of pCas-*ΔACH1*, pCas-*ΔTES1*, *pCas-ΔEHD3* plasmid |
| gRNA-r | aaaGGTCTCaAAACCTAGACACAGGGTAATAACTGATATAATTAAATTGAAGCTC |  |
| gTES1-f | aaaGGTCTCAGATCCTCGTCGCCCGCGTGCTCCAGTTTTAGAGCTAGAAATAGCAAGTTA |  |
| gEHD3-f | aaaGGTCTCAGATCACCAATGTCCATCTCGGGCAGTTTTAGAGCTAGAAATAGCAAGTTA |  |
| donor-ACH1-f | AGGAAGCTCACGAGCTTATTCCATTGTTCAAGAATGGTCAGTACCTTGGGTGGTCCGGTTTGGTCACTGA | Donor of *ΔACH1* |
| donor-ACH1-r | CACGGGCTCTTTCCTTAGGCGATAGACCTCTTAGATCCGCCAAACCTTGGTCAGTGACCAAACCGGACCA |  |
| donor-TES1-f | AATATTGGAACTGGTTCCTCTTTCGCCTACCAGTTTTGTCACAAAGTATCTGCCTGCCGCAC TGGGACAA | Donor of *ΔTES1* |
| donor-TES1-r | CCATGATGCGTCCCGACTGTGTGTCGAAATACTTGCCCTGTACGAGGTGCTTGTCCCAGTGCGGCAGGCA |  |
| donor-EHD3-f | TACCACACGTACATTCATGACTACTCAACCCCAGCTAAATGTCACCGACGCACCACCTGTCGAAAGAGAA | Donor of *ΔEHD3* |
| donor-EHD3-r | CCCAGTTTCCCTCTTCTAGAAGGAATCACATTGGCAAAGTGATTTAGTACTTCTCTTTCGACAGGTGGTG |  |

**Table S4** Enzymatic properties of MCLs

| **Enzyme** | **Organism** | **Reactions** | **Commentary** | **Specific activity [µmol/min/mg]^a^** | **Condensation/lyase activity ratio** |
| --- | --- | --- | --- | --- | --- |
| *Ca*MCL | *Chloroflexus aurantacus* | L-malyl-CoA ⇄ Acetyl-CoA + Glyoxylate | Cleavage of L-malyl-CoA | 2.3 | 0.2 |
|  |  |  | Acetyl-CoA condensation | 0.48 |  |
|  |  | β-methylmalyl-CoA ⇄ Propionyl-CoA + Glyoxylate | Cleavage of β-methylmalyl-CoA | 6.5 | 0.1 |
|  |  |  | Propionyl-CoA condensation | 0.5 |  |
| *Rs*MCL | *Rhodobacter sphaeroides* | L-malyl-CoA ⇄ Acetyl-CoA + Glyoxylate | Cleavage of L-malyl-CoA | 4.1 | 3.4 |
|  |  |  | Acetyl-CoA condensation | 14 |  |
|  |  | β-methylmalyl-CoA ⇄ Propionyl-CoA + Glyoxylate | Cleavage of β-methylmalyl-CoA | 4.5 | 4.4 |
|  |  |  | Propionyl-CoA condensation | 20 |  |
| *Rc*MCL | *Rhodobacter capsulatus* | L-malyl-CoA ⇄ Acetyl-CoA + Glyoxylate | Cleavage of L-malyl-CoA | 18 | 2.1 |
|  |  |  | Acetyl-CoA condensation | 37 |  |
|  |  | β-methylmalyl-CoA ⇄ Propionyl-CoA + Glyoxylate | Cleavage of β-methylmalyl-CoA | 5.7 | - |
|  |  |  | Propionyl-CoA condensation | - |  |

**Reference:**

1. Mattozzi M, Ziesack M, Voges MJ, et al. Expression of the sub-pathways of the Chloroflexus aurantiacus 3-hydroxypropionate carbon fixation bicycle in *E. coli:* Toward horizontal transfer of autotrophic growth. *Metabolic Engineering*. **2013**;*16*:130–139.

2. Zhang Y, Su M, Qin N, et al. Expressing a cytosolic pyruvate dehydrogenase complex to increase free fatty acid production in *Saccharomyces cerevisiae*. *Microb Cell Fact*. **2020**;*19(1)*:226.

3. Zhang Y, Wang J, Wang Z, et al. A gRNA-tRNA array for CRISPR-Cas9 based rapid multiplexed genome editing in *Saccharomyces cerevisiae*. *Nat Commun*. **2019**;*10(1)*:1053.

4. Jessop-Fabre MM, Jakočiūnas T, Stovicek V, et al. EasyClone-MarkerFree: A vector toolkit for marker-less integration of genes into *Saccharomyces cerevisiae* via CRISPR-Cas9. **2016**;*11(8)*:1110-1117.
